# Unveiling Differences in Root Defense Mechanisms Between Tolerant and Susceptible Olive Cultivars to *Verticillium dahliae*

**DOI:** 10.3389/fpls.2022.863055

**Published:** 2022-04-25

**Authors:** Martina Cardoni, Carmen Gómez-Lama Cabanás, Antonio Valverde-Corredor, Rafael Villar, Jesús Mercado-Blanco

**Affiliations:** ^1^Department of Crop Protection, Institute for Sustainable Agriculture (CSIC), Córdoba, Spain; ^2^Department of Botany, Ecology and Plant Physiology, University of Córdoba, Córdoba, Spain

**Keywords:** functional traits, host defense response, lignin content, *Olea europaea*, olive root architecture, root membrane permeability, specific root length, Verticillium wilt

## Abstract

Verticillium wilt of olive (VWO), caused by the soil-borne vascular pathogen *Verticillium dahliae*, is one of the most devastating diseases affecting olive, the woody crop. One of the best VWO management measures is the use of tolerant cultivars. Yet, our knowledge about defense mechanisms that operate at the root level to explain tolerance to this disease is incomplete. Moreover, most of the approaches so far followed focus only on a specific mechanistic level (e.g., genetic, physiological, or biochemical) rather than on a holistic/multilevel perspective. In this study, eighteen root functional traits, the time-course expression of nine defense-related genes, the root lignin content, and the root membrane permeability were evaluated in six olive varieties differing in their level of tolerance/susceptibility to VWO. The aim was to find links between the level of tolerance to VWO and specific root defense mechanisms at the structural, genetic, biochemical, and physiological levels. Tolerant and susceptible cultivars showed substantial differences in the root system architecture and root lignin content. VWO-susceptible cultivars presented roots with higher specific length and area, but lower diameter and larger number of forks and tips compared to tolerant varieties that also showed less branched roots, higher root diameter, and larger basal content of lignin. Interestingly, VWO-tolerant varieties significantly increased their root lignin content and root membrane permeability after inoculation with *V. dahliae*. These results were seldom (or not at all) observed in the susceptible plants. At the genetic level, genes related to defense mechanisms, such as cell wall lignin biosynthesis (*C4H* and *CO-MT*), production of hydrolytic enzymes able to degrade the fungal cell wall (β*-1.3-glucanase*), and activation of innate immunity (*BAK1* and *WRKY5*) increased their expression in tolerant cultivars from early moments after inoculation, in contrast to the susceptible ones. These results showed that differences in the root system architecture and lignin content may greatly determine the performance of olive against colonization and invasion by *V. dahliae*. Moreover, the increase in root membrane permeability in the presence of the pathogen was a typical response of tolerant cultivars. Finally, VWO-tolerant cultivars were able to mount a more intense and rapid defense-related genetic response to respond to the attack by *V. dahliae*.

## Introduction

Verticillium wilt of olive (*Olea europaea* L.) (VWO), caused by the soil-borne pathogenic fungus, *Verticillium dahliae* (division *Ascomycota*, family *Plectosphaerellaceae*) (Inderbitzin et al., 2011), is a major concern in many olive-growing countries. There are a number of factors that make it difficult to control the disease (Tsror, 2011), and the implementation of an integrated disease management strategy is thus highly recommended (López-Escudero and Mercado-Blanco, 2011; Montes-Osuna and Mercado-Blanco, 2020). For this approach, the use of resistant/tolerant cultivars is the most efficient and economically plausible measure for reducing the severity and the spread of the disease (Garcia-Ruiz et al., 2014; Arias-Calderón et al., 2015; Valverde et al., 2021). Although no olive cultivar has so far been reported as fully resistant to VWO, some varieties have shown moderate/high level of tolerance; that is, they are able to cope with the pathogen infection without developing evident symptoms of the disease (Robb, 2007; Markakis et al., 2009; Ramírez-Tejero et al., 2021).

Plant tolerance is determined by a combination of constitutive (prior to infection) and inducible (during pathogen attack) defense mechanisms (Thatcher et al., 2005). In the particular case of VWO, several studies have demonstrated that the first defense mechanisms against *V. dahliae* take place in the roots (Gómez-Lama Cabanás et al., 2015; Gharbi et al., 2017a; Leyva-Pérez et al., 2018). Passive defenses are the first obstacle that a pathogen has to overcome before the onset of the disease. The typical preformed, basal defenses are morphological and structural barriers, including cell walls reinforced with callose, lignin, and suberin (Ferreira et al., 2007; Vanholme et al., 2010; Lee et al., 2019). In some plant species, a relation between root morphology and pathogen tolerance has been found. For instance, Dias et al. (2004) showed a correlation between the lateral branching of the root structure (length and number) of melon plants and their resistance to the soil-borne fungus, *Monosporascus cannonballus*. An association between the root branching architecture and Fusarium rot resistance has also been demonstrated in common beans (Román-Avilés et al., 2004). For olive plants, limited information is yet available about belowground defense mechanisms, including root anatomy and structure, and tolerance to *V. dahliae* (Leyva-Pérez et al., 2018). Bubici and Cirulli (2012) demonstrated that the tolerances of olive cultivars to VWO and low-symptoms severity were related to the ability of the plant to produce vascular plugging. Concerning the olive root system structure, Chatzistathis et al. (2013), observed differences among Greek olive varieties. Indeed, they reported that cultivar Koroneiki, classified as VWO tolerant (Markakis et al., 2010), presented less lateral branching and root hairs compared to other varieties (Kothreiki and Chondrolia Chalkidikis) that were qualified as susceptible to the disease. Likewise, we recently found clear differences in specific olive functional traits in the leaves (e.g., area, dry matter content), entire plant (e.g., leaf and stem mass fraction), and roots (e.g., root-specific area and length) that are related to the level of tolerance of olive cultivars to *V. dahliae* (Cardoni et al., 2021). However, there are no studies about the specific structure of olive root branching and the susceptibility to this pathogen.

In addition to the root system architecture, the layer composed of suberin and lignin deposition is an important mechanical defense barrier preventing the entry of soil-borne pathogens. Suberin is a heteropolymer deposited and immobilized in the plant cell wall, constituting one of the main components of the tree bark. The suberization process supplies impermeability to the well-structured necrophylactic periderm, a post-infection mechanism of the defense developed against pathogens in the woody broadleaves and conifers (Danti et al., 2018). Lignin is a major phenolic polymer that constitutes the secondary cell wall in vascular plants, providing strength and resistance to the plants (Lee et al., 2019). Gharbi et al. (2017b) found higher constitutive lignin content in the roots and stems of a *V. dahliae*-tolerant olive variety compared to a susceptible one prior to be infected by the pathogen. Interestingly, different post-infection content of lignin has also been reported in the stems of olive cultivars differing in susceptibility to another vascular pathogen (i.e., the bacterium *Xylella fastidosa*) (Sabella et al., 2018). If these mechanical defenses are overcome, elicitors produced by the pathogen induce a cascade of defense responses in the plant cell that include cell wall thickening with further deposition of lignin and suberin (Ferreira et al., 2007), generation of salicylic acid (SA) at the site of infection and distal tissues triggering systemic acquired resistance (SAR) (Narváez et al., 2020), acceleration of cell death (hypersensitive response), production of reactive oxygen species (ROS) (Mittler et al., 2004), phenolic compounds (Gharbi et al., 2017b), pathogenesis-related (PR) proteins, and many other defense-related proteins (Thatcher et al., 2005; Balasubramanian et al., 2012).

Plant hormones are key elements in regulating defense responses against abiotic and biotic stresses. In particular, jasmonic acid (JA), ethylene (ET), and SA play a central role in the activation of plant defense mechanisms against pathogens. The first two phytohormones are mainly involved in the induction of innate systemic resistance, while the SA pathway is activated at an early stage of infection by the induction of SAR at the site of infection, which prevents the spread of the pathogen (Gharbi et al., 2017a). The importance of SA/SAR was studied in olive plants, and the accumulation of the PR1 (antifungal), PR2 (β-1,3-glucanases), and PR5 (thaumatin-like) proteins was confirmed (Narváez et al., 2020). Crosstalk between SA and JA, which is fundamental in defense signaling, has been well documented in plants. However, how this dialogue takes place, and the components involved still remain to be fully elucidated. The WRKY transcription factors have been reported as required for JA and SA pathways crosstalk (Gupta et al., 2000). Indeed, a recent study identified the *WRKY70* gene as an activator of SA-induced genes and a repressor of JA-responsive genes in *Arabidopsis thaliana* inoculated with the phytopathogenic bacterium *Erwinia carotovora* (Li et al., 2004). In olive, different *WRKYs* (i.e., *WRKY44, WRKY33*, and *WRKY20*) were systemically upregulated in the leaves and stems of the VWO-tolerant cultivar Frantoio upon inoculation with *V. dahliae*. Furthermore, *WRKY5* was earlier identified as potentially involved in defense response at the root level. Indeed, this transcription factor-coding gene was upregulated in olive roots upon colonization by *Pseudomonas simiae* PICF7, a biological control agent against VWO (Schilirò et al., 2012).

Reactive oxygen species has a dual role in plant biology; as these molecules are toxic byproducts of the aerobic metabolism and key regulators of growth and defense pathways (Noctor et al., 2018). The function of ROS as signaling molecules in plant cells suggests that during the course of evolution, plants were able to achieve a high degree of control over ROS toxicity (Mittler et al., 2004). Indeed, plants developed an efficient strategy to maintain ROS homeostasis at various sites of their cells, based on antioxidant systems composed of redox-controlling enzymes (Chen et al., 2015; de Pascali et al., 2019). The best-known enzymes that cooperate to protect olive cells from ROS toxicity are peroxidase (POX), polyphenol oxidase (PPO), ascorbate peroxidase (APX), polyphenol oxidase (POD), catalase (CAT), and superoxide dismutase (SOD) (Mittler et al., 2004; Gharbi et al., 2017b; de Pascali et al., 2019). In addition to the antioxidative role, these enzymes participate in an integrated plant defense response to a variety of stresses through cell wall toughening and by the production of toxic secondary metabolites. Gharbi et al. (2017b) showed that the early upregulation of *POX* and *PPO* in *V. dahliae*-infected olive cultivars was related to enhanced ability to limit the invasion and spread of the pathogen through the synthesis of polyphenols and lignin accumulation at the site of infection. Moreover, Leyva-Pérez et al. (2018) reported differential expression patterns of six genes with a strong response to ROS stress in tolerant (“Frantoio”) and susceptible (“Picual”) cultivars upon *V. dahliae* inoculation.

Pathogens can eventually overcome the above-mentioned defense mechanisms by producing effectors to activate the so-called effector-triggered immunity (Bari and Jones, 2009). In the particular case of the olive and *V. dahliae* interaction, Leyva-Pérez et al. (2018) showed that the gene *BAK1*, a positive regulator of pathogen-associated molecular patterns (PAMPs), may be involved in the initiation of innate immunity in olive plants. These authors related the downregulation of *BAK1* in “Picual” plants (VWO-susceptible) with the lower capacity of this cultivar to cope with *V. dahliae* infection compared to that displayed by “Frantoio” (VWO-tolerant).

Differences in the root architecture, lignin content, and defense mechanisms activation may determine the performance of olive cultivars against colonization and invasion by *V. dahliae*. While root anatomy of some species has been studied from both genetic and functional points of view (Wachsman et al., 2015), the information regarding the olive root system and its relationship with tolerance/susceptibility to *V. dahliae* is yet scarce. Considering the previous information, this study aims to unravel distinctive belowground defense mechanisms contributing to the tolerance and susceptibility of olive cultivars to VWO at four different levels: (i) structural, by analyzing root functional traits; (ii) genetic, by assessing the time-course expression of selected genes coding for proteins involved in the lignin biosynthesis pathway (*C4H* and *CO-MT*), ROS response (*APX, POX*), PR-mediated defense response (i.e., chitinase, β-1,3-glucanase) as well as the disease resistance-responsive protein (DRR2), the brassinosteroid insensitive 1-associated receptor kinase gene (*BAK1*), and the SA signal transduction factor, *WRKY5*; (iii) biochemical, by quantifying root lignin content; and (iv) physiological, by assessing the root membrane permeability. The hypotheses to be tested are as follows: (i) root branching architecture explains the tolerance/susceptibility of olive cultivars to *V. dahliae*, and (ii) the timing and intensity of genetic defense responses explain the VWO tolerance level of olive cultivars.

## Materials and Methods

### Plant Material

Six olive varieties were chosen for this study, three of them classified as tolerant (Frantoio, Empeltre and Changlot Real) and the other three as susceptible (Picual, Hojiblanca, and Lechín de Sevilla) to VWO (López-Escudero et al., 2004; Trapero et al., 2012, 2015, 2018; Garcia-Ruiz et al., 2014; Gómez-Lama Cabanás et al., 2015; Serrano et al., 2021; Valverde et al., 2021). Five-month-old olive plants contained in polyvinyl chloride (PVC) pots (11 x 11 x 12 cm) filled with a peat-based substrate containing the universal slow-release fertilizer Osmocote Exact standard 12–14 M (1g/L) (ICL Specialty Fertilizers-Iberia, Murcia, Spain) and calcium carbonate (0.5 g/L), with a final pH of 7.5 ± 0.5, were purchased in a commercial nursery located in Córdoba province (southern Spain). Before starting the experiments, the plants were acclimated in a greenhouse for 2 months under natural lighting at a temperature of 23 ± 4°C, and relative humidity ranging from 40% (day) to 80% (night).

Olive plants were used in two different experiments. On the one hand, a bioassay was carried out to evaluate the differences in root functional traits of tolerant vs. susceptible cultivars to *V. dahliae*. On the other hand, an experiment was designed to conduct a time course of the expression of nine selected olive genes in roots upon inoculation with *V. dahliae* (defoliating [D] pathotype). Moreover, membrane permeability and lignin content in the roots of this set of plants were determined in the absence/presence of the pathogen (refer to Section Root Tissues Manipulation and Functional Traits Measurement).

### Root Tissues Manipulation and Functional Traits Measurement

Two months after the acclimation period, the olive plants were transplanted into bigger PVC pots (3l volume), filled with the same substrate described above, to allow for the maximum, unconstrained growth of the root system. After 3 months of growth in these pots, four plants per variety were sampled. Each plant was carefully uprooted from the pot, and their root systems were manipulated as previously described (Cardoni et al., 2021). For each sample, the root system was divided into two halves. One half was entirely used to study the functional traits of thick (diameter > 2 mm) and thin (<2 mm) roots. The other half was used for the analysis of the functional traits of root branching portion. For this purpose, two intact roots branching, from the basal root to the outermost ramifications, were chosen to represent the root system of the sample. These roots were selected and analyzed without modifying or altering the branching structure. All root segments were placed on a scanner (Epson Expression 164, Seiko Epson Corp. Nagano-Ken, Japan) in a transparent plastic tray filled with water and analyzed using WinRHIZO Pro v.3.10b (Regent Instrument Inc., Quebec, Canada). The above software enabled to measure the following parameters: mean root diameter (mm), total length (cm), surface area (cm^2^), root volume (cm^3^), length of each diameter class (between 0 and 4.5 mm), and the number of root tips, forks, and crossings. Finally, the entire sample was used to estimate the fresh and dry (70°C, 48 h) masses of the root system. Based on all these measurements, different functional traits were calculated (Table 1).

**Table 1:** Results of ANOVA analyses for different root traits using the tolerance level to Verticillium wilt (“tolerance”) or olive variety (“variety”) as factors.

**Root tipe**	**Trait**	**Abbreviation**	**Units**	**Explained variance by “tolerance” (%)**	**Explained variance by “variety” (%)**
Thin root	Specific root length Thin	SRL Thin	cm g^−1^	87.0^***^(–)	**60.5** ^**^
	Root tissue density Thin	Density Thin	g cm^−3^	61.1	25.4
	Root average diameter Thin	AvgDiam Thin	cm	86.2^***^(+)	**52.1** ^*^
	Specific root area Thin	SRA Thin	cm^2^ g^−1^	91.1^***^(–)	**67.4** ^*^
	Root dry matter content Thin	RDMC Thin	g g^−1^	69.4	51.3
Thick root	Specific root length Thick	SRL Thick	cm g^−1^	80.4^**^(–)	**68.1** ^**^
	Root tissue density Thick	Density Thick	g cm^−3^	28.6	32.9
	Root average diameter Thick	AvgDiam Thick	cm	0.01	51.2
	Specific root area Thick	SRA Thick	cm^2^g^−1^	1.9	46.1
	Root dry matter content Thick	RDMC Thick	g g^−1^	7.4	**67.2** ^**^
Branching root portion	Specific root length Branching portion	SRL Branching	m g^−1^	78.5^*^(–)	**60.7** ^**^
	Root tissue density Branching portion	Density Branching	g cm^−3^	17.5	13.7
	Root average diameter Branching portion	AvgDiam Branching	cm	5.1	**86.7** ^**^
	Specific root area Branching portion	SRA Branching	cm^2^ ^−1^	92.4^***^(–)	**74.9** ^**^
	Root dry matter content Branching portion	RDMC Branching	g g^−1^	12.7	45.6
	Number of root tips	Tips	-	93.9^***^(–)	91.62^***^
	Number of root forks	Forks	-	87.9^**^(–)	95.23^***^
	Number of root crossing	Crossings	-	90.0^***^(–)	82.2^***^

Three different types of roots were selected to measure the functional traits. On the one hand, thin (diameter < 2 mm) or thick (diameter > 2 mm) roots were selected. On the other hand, functional traits for the branching root portion were measured on intact root branching, from the basal root to the outermost ramifications, representing the sample root system. The explained variance percentage by the “tolerance” (fifth column) or “variety” (sixth column) factors (100 × SS factor/SS total) and the level of significance carried out with ANOVA analysis (^*^, p < 0.05; ^**^, p < 0.01; ^***^, p < 0.001) are shown (significant values are in bold type). SS represents the sum of squared differences from the mean. Considering the “tolerance” factor, (+) means higher value for tolerant and (–) means lower value for this trait.

### Inoculation With *Verticillium dahliae* and Plant Tissue Manipulation to Assess the Expression of Olive Genes in Roots

After the acclimation period (refer to Section Plant Material), the olive plants were inoculated with *V. dahliae* V937I, an isolate representative of the D pathotype (Collado-Romero et al., 2006), by adding 150 ml of conidia suspension, per pot, (5·10^6^ conidial/ml) prepared as described by the method of Gómez-Lama Cabanás et al. (2017). Non-inoculated (control) plants were watered just with 150 ml of tap water. At time-point 0, and at 1, 2, 7, and 15 days after inoculation (DAI), 18 plants per variety (i.e., nine *V. dahliae*-inoculated and nine control plants) were sampled. The root system of each plant was uprooted and gently cleaned under tap water. Then, the roots of three *V. dahliae*-inoculated and three control plants per time-point were rapidly frozen in liquid nitrogen and stored at −80°C until being used for RNA extraction. Of the remaining plants, the roots of three of them (kept at room temperature) were used to perform membrane permeability measurement and the roots of the other three were rapidly frozen in liquid nitrogen and stored at −80°C until being used to determine the lignin content.

Additionally, seven *V. dahliae*-inoculated and three control (non-inoculated) plants per variety were used to evaluate the disease development by scoring the severity of symptoms on a 0–4 scale, according to the percentage of affected leaves and twigs (0, no symptoms; 1, 1–33%; 2, 34–66%; 3, 67–100%; 4, dead plants). Scoring of symptoms was performed twice a week during 100 days after pathogen inoculation. The area under the disease progress curve (AUDPC) and the disease severity (S) were calculated, and the analysis of variance (ANOVA) for these parameters was carried out as described earlier (Maldonado-González et al., 2015). Parameters, such as the disease incidence (DI), mortality (M), and disease intensity index (DII), were also determined according to the method of Mercado-Blanco et al. (2003).

### *In planta* Detection of *Verticillium dahliae*

The presence of *V. dahliae* in inoculated plants was confirmed according to the procedure described by Mercado-Blanco et al. (2003) with some modifications. Briefly, total DNA was extracted from the root tissues of control and inoculated plants (three plants per treatment) sampled at 7 and 15 DAI. The DNA was purified using the Maxwell RSC Pure Food GMO and Authentication Kit (Promega Corporation, Madison, WI, USA) following the manufacturer's instructions. Nested PCR was carried out using primers for the specific detection of the D pathotype. The primer pair DB19/DB22 was used in the first round of amplification while primer pair DB19/espdef01 was used in the second round. The presence of V937I in root samples was confirmed by the amplification and visualization of a 330-bp PCR product in 1% of agarose gel (Mercado-Blanco et al., 2003).

### RNA Extraction, Purification, and CDNA Synthesis

Total RNA was extracted from the olive roots sampled at 0, 1, 2, 7, and 15 DAI. The RNA extraction was performed using the RNeasy Plus Mini Kit (Qiagen, Hilden, Germany), according to the manufacturer's instructions, and adding polvinylpyrrolidone 40,000 (VWR International, Radnor, PA, USA) to the lysis buffer at a final concentration of 5 mg ml^−1^. A motorized pestle mixer (VWR^®^ Pellet Mixer, VWR, Intl., Radnor, PA, USA) was used for the homogenization step. Samples were treated with TurboTM DNase I (Life Technology, Invitrogen Carlsbad, CA, USA) for 45 min at 37°C followed by a phenol-chloroform/ethanol purification process to eliminate traces of genomic DNA (gDNA). Real-time qPCR was performed using DNase-treated RNA as a template and primers for the 60S ribosomal protein L18-3 (60S RBP L18-3) genomic sequence (Supplementary Table 1) were used to check for the absence of gDNA. Only samples with undetected amplification after 40 cycles were further used. RNA yield and quality (A260/230, A260/280) were determined using Nanodrop ND-1000TM spectrophotometer (Thermo Fisher Scientific, Wilmington, DE, USA) and Qubit 3.0 fluorometer (Life Technologies, Grand Island, NY, USA). Synthesis of cDNA was performed from 400 ng of total RNA by using an oligo(dT)18 primer and the RevertAid H Minus First Strand cDNA Synthesis kit (Fisher Scientific, Inc., Waltham, MA, USA) following the manufacturer's instructions except that the first incubation was performed at 70°C instead of 65°C, and the reaction stop was extended for five additional minutes.

### Real-Time qPCR Primers and Time-Course Gene Expression Analyses

Nine olive genes related to the lignin biosynthetic pathway (*C4H* and *CO-MT*), plant defense responses (*chitinase*, β*-1,3-glucanase, BAK1*, and *DRR2*), ROS response (*APX* and *POX*), and SA signaling transduction pathway (*WRKY5*) were selected to examine their expression patterns in olive roots at five time-points (0, 1, 2, 7, and 15 DAI) (Supplementary Table 1). Primer 3 software version 0.4.0 (http://primer3.ut.ee/) and the Oligo Analyzer tool (Integrated DNA Technologies, Inc., Coralville, IA, USA) were used to design the most suitable primers. All primers (Supplementary Table 1) were custom ordered from a commercial supplier (Creative Biogene, Shirley, NY, USA) and tested at 100 nM by PCR with gDNA.

Real-time qPCR (RT-qPCR) runs were performed in a Bio-Rad CFX 384 RT-qPCR detection system (Bio-Rad Laboratories Inc., Hercules, CA, USA). Relative expression for each studied gene was repeated at least once for each biological replicate in independent RT-qPCR assays (plates), and three technical replicates per sample and per plate were always included. The design of RT-qPCR experiments, calculations, and statistics used followed the MIQE guidelines (Bustin et al., 2009). Each RT-qPCR reaction contained 2 μL of a 20x diluted template cDNA, 0.3μM of each primer, 5 μL of SYBR® Green Supermix (BioRad), and distilled water up to a total volume of 10 μL. λ-DNA (5 ng ml^−1^) was added to the cDNA samples as carrier DNA to minimize the absorption and Poisson effects.

All RT-qPCRs were run as follows: at 95°C for 10 s, 39 cycles at 95°C for 15 s, 55/57/60°C (depending on the optimal annealing temperature of each primer pair used) for 30 s, and 72°C for 15 s (refer to Supplementary Table 1). Standard curves were generated for each selected gene using five serial four-fold dilutions of sample-pooled cDNA to calculate gene-specific amplification efficiencies (E), correlation coefficients (r), and the corresponding linear equations (Supplementary Table 1). Data resulting from RT-qPCR were normalized to the elongation factor 1α (*EF1-* α) and 60S ribosomal protein L18-3 (*60S RBP L18-3*) genes (Ray and Johnson, 2014). To determine the gene expression level quantification cycles (Cq), the values were converted into calibrated normalized relative quantities (CNRQ), using the Biorad CFX Maestro software integrated in the thermal cycler Bio-Rad CFX 384.

### Quantification of Acid-Insoluble, Acid-Soluble, and Total Lignin Contents in Olive Roots

The lignin content of olive roots was determined at three time points (0, 7, and 15 DAI). Olive root tissues stored at −80°C were ground into a fine powder in liquid nitrogen and dried at 105°C for 24 h. Samples of 0.3 g of the dry material were used. The acid-insoluble residue (AIR) was determined gravimetrically while the acid-soluble lignin (ASL) was determined by spectrophotometry as previously described (Cara et al., 2008; Cardoni et al., 2021). Total lignin content was calculated by the sum of AIR and ASL.

### Assessment of Root Membrane Permeability

Changes in the root membrane permeability due to the presence of *V. dahliae* were monitored using fresh roots of three different plants collected at different time points (0, 1, 2, 7, and 15 DAI), both for *V. dahliae*-inoculated and control (non-inoculated) plants. Each root sample, after a quick cleaning with distilled water to remove potting substrate which could affect the measurement, was cut into small pieces (1 cm long approximately) and incubated overnight in 25 mL of sterile distilled water at room temperature (Gharbi et al., 2017b). Root samples were removed from the water that was then used to measure the permeability (three replicates per treatment) using a pH/conductivity meter (model CCMD510 WPA, Thermo Fisher Scientific Inc., Göteborg, Sweden). Values were scored in micro Siemens (μS) at 25°C.

### Statistical Analysis

To investigate the possible differences between VWO-tolerant and VWO-susceptible cultivars, a one-way ANOVA analysis, conducted with the R function *aov*, was performed separately with data of functional traits, relative gene expression, lignin content, and membrane permeability. Afterward, significant differences were evaluated with the Tukey's *post-hoc* test with a *p* level of 0.05 (R package *agricolae*) (De Mendiburu and Simon, 2015). The Principal Component Analysis (PCA), using the R package *factoextra* (Kassambara, 2016), was carried out on functional traits data to assess the most influential variables on tolerant and susceptible olive varieties. For this analysis, only the variables with a contribution higher than 5% for the two PCA dimensions were considered. All statistical analyses were performed using the statistical software, R (R. Team, 2015).

## Results

### Evaluation of VWO Symptoms

“Frantoio”, “Empeltre”, and “Changlot Real” plants did not show any visible symptoms after 100 DAI. “Picual” plants were the first to show symptoms (leaf rolling, wilting, and defoliation) at 31 DAI, followed by “Lechín de Sevilla” (46 DAI) and “Hojiblanca” (52 DAI). No symptoms of VWO were observed in control plants at any time. “Picual” showed the highest values in all the disease parameters evaluated, followed by “Lechín de Sevilla,” although no plant of this cultivar died. In contrast, “Hojiblanca” plants showed the lowest values of AUDPC, DI, DII, and S, but more than 14% of the plants were dead at the end of the experiment (Table 2). Finally, nested PCR assays confirmed the absence of the pathogen in the roots of control plants, while *V. dahliae* was detected in all analyzed roots of pathogen-inoculated “Frantoio”, “Empeltre”, “Changlot Real,” and “Picual” plants at 7 DAI and in “Lechín de Sevilla” and “Hojiblanca” roots at 15 DAI (Table 2).

**Table 2:** Verticillium wilt of olive parameters scored for the six olive varieties under study.

**Variety**	**S**	**DI**	**M**	**DII**	**AUDPC**	**Presence^**a**^ of *Vd* in roots of inoculated plants at 7 DAI**	**Presence^**a**^ of *Vd* in roots of inoculated plants at 15 DAI**
Picual	3.93 a	100	71.43	0.98	165.46 a	+	+
Hojiblanca	1.14 b	57.14	14.29	0.28	34.03 b	–	+
Lechín de Sevilla	1.46 b	100	0	0.36	48.52 b	–	+
Frantoio	0.0 c	0.0	0.0	0.0	0.0 c	+	+
Changlot	0.0 c	0.0	0.0	0.0	0.0 c	+	+
Empeltre	0.0 c	0.0	0.0	0.0	0.0 c	+	+

*Olive plants were inoculated with Verticillium dahliae V937I, an isolate representative of the defoliating pathotype. Values correspond to disease severity index (ranging 0–4) (**S**), disease incidence (%) (**DI**), dead plants at the end of the experiments (%) (**M**), disease intensity index (ranging 0–1) (**DII**) calculated with data on incidence and severity of symptoms, and area under the disease progress curve (**AUDPC**). Data are the average of three randomly distributed blocks each with four pots (plants) per treatment. In **S** and **AUDPC** columns, values followed by different letters are significantly different according to Tukey's post hoc test (p < 0.05). All disease parameters were calculated at the end of the experiments (100 days after inoculation; DAI)*.

a *The presence/absence of V. dahliae DNA (Vd) in the roots was assessed by nested PCR at 7 and 15 DAI (see main text for details). +, presence of the pathogen in at least two out of the three plants sampled; –, absence of the pathogen in the three sampled plants*.

### Tolerant and Susceptible Olive Cultivars to Verticillium Wilt Show Differences in Root Functional Traits

**Figure 1:**
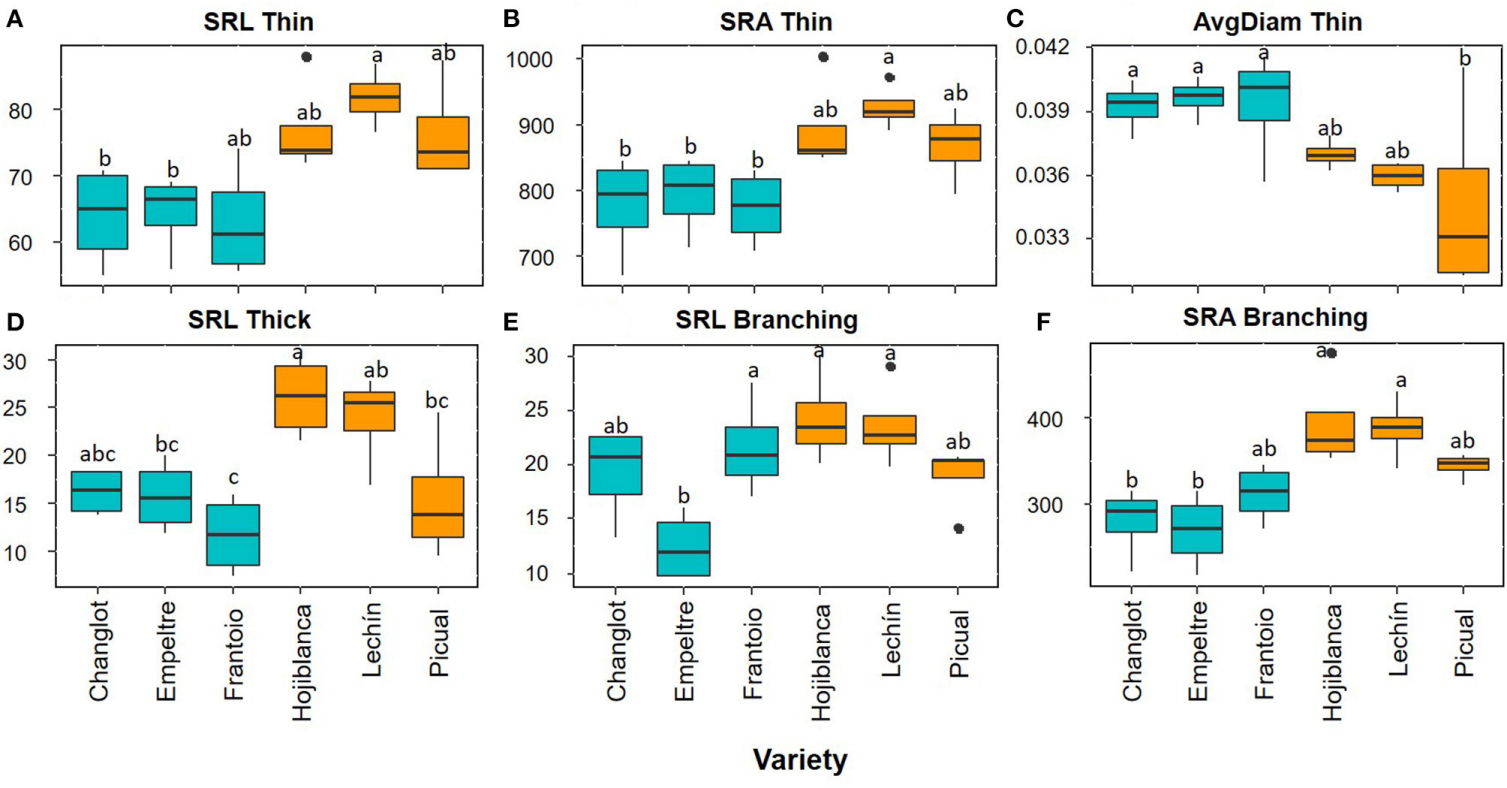
Box plots showing median values (*n* = 4) of SRL thin (“specific root length thin”) (cm g-1) **(A)**, SRA thin (“specific root area thin”) (cm^2^ g^−1^) **(B)**, AvgDiam thin (“root average diameter thin”) (cm) **(C)**, SRL thick (“specific root length thick”) (cm g^−1^) **(D)**, SRL Branching (“specific root length branching portion”) (cm g^−1^) **(E)**, and SRA branching (“specific root area branching portion”) (cm^2^ g^−1^) **(F)**. Tolerant cultivars are represented in light blue while the susceptible ones are shown in orange. Letters indicate Tukey's *post hoc* tests at the *p* < 0.05 level, following ANOVA.

**Figure 2:**
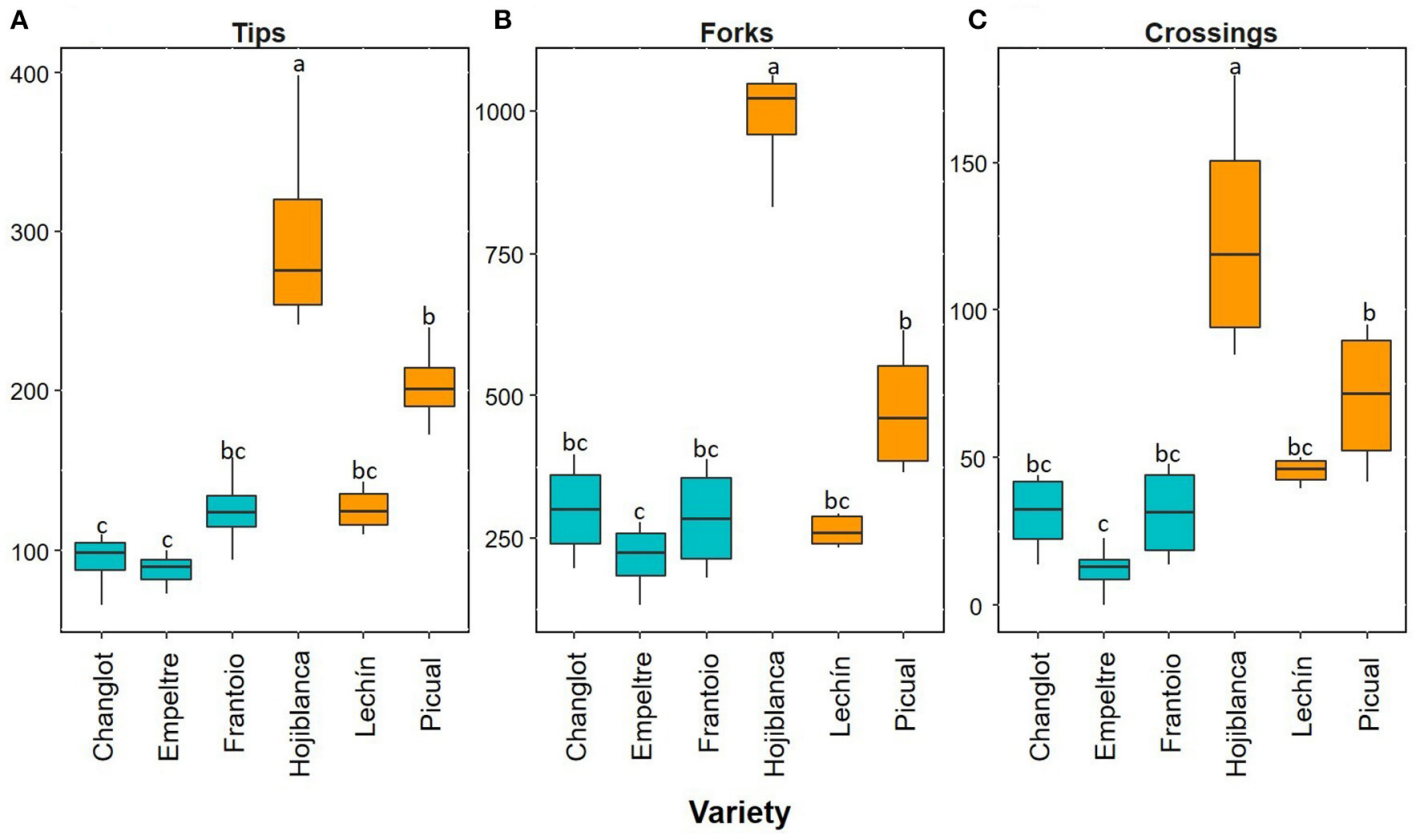
Box plots showing median values (*n* = 4) of number of root tips **(A)**, forks **(B)**, and crossings **(C)**. Tolerant cultivars are represented in light blue while the susceptible ones are shown in orange. Letters indicate Tukey's *post hoc* tests at the *p* < 0.05 level, following ANOVA.

Overall, the root functional traits examined here showed significant differences between tolerant and susceptible cultivars, and also among the six varieties (Table 1). Regarding thin roots, ANOVA analysis showed significant differences for specific root length (SRL), specific root area (SRA), and average diameter (AvgDiam), which explained the high percentage of variance between VWO-tolerant and VWO-susceptible cultivars (87, 91.1, and 86.2%, respectively). The first two traits (SRL and SRA Thin) presented larger values for the susceptible cultivars, especially “Lechín de Sevilla” (Figures 1A,B). In contrast, higher AvgDiam values were scored for the tolerant varieties, which were significantly different (*p* < 0.05) to that of “Picual” plants that exhibited the lowest diameter (Figure 1C). When considering thick roots, only SRL showed significant differences between the tolerant and susceptible varieties, explaining the greatest percentage of variance (80.4%). This functional trait generally showed higher values in the susceptible cultivars, particularly in “Hojiblanca” plants that exhibited significant differences (*p* < 0.05) compared to “Empeltre” and “Frantoio” (Figure 1D). Regarding the functional traits of the root branching portion, SRL, SRA, tips, forks, and crossings showed significant differences between the tolerant and susceptible varieties, explaining the large percentage of variance (between 78 and 93%) (Table 1). The susceptible cultivars showed a general trend to display higher SRL and SRA values, mainly “Hojiblanca” and “Lechín de Sevilla” that showed significant differences (*p* < 0.05) with “Empeltre” (SRL and SRA) and “Changlot Real” (SRA) (Figures 1E,F). It is worth mentioning the differences found in the number of tips, forks, and crossings, which mostly showed higher values in susceptible cultivars. This was particularly true in the case of “Hojiblanca” and “Picual” roots which for instance, presented significant differences (*p* < 0.05) with “Empeltre” for all these traits, and with “Changlot Real” for the number of tips (Figures 2A–C).

**Figure 3:**
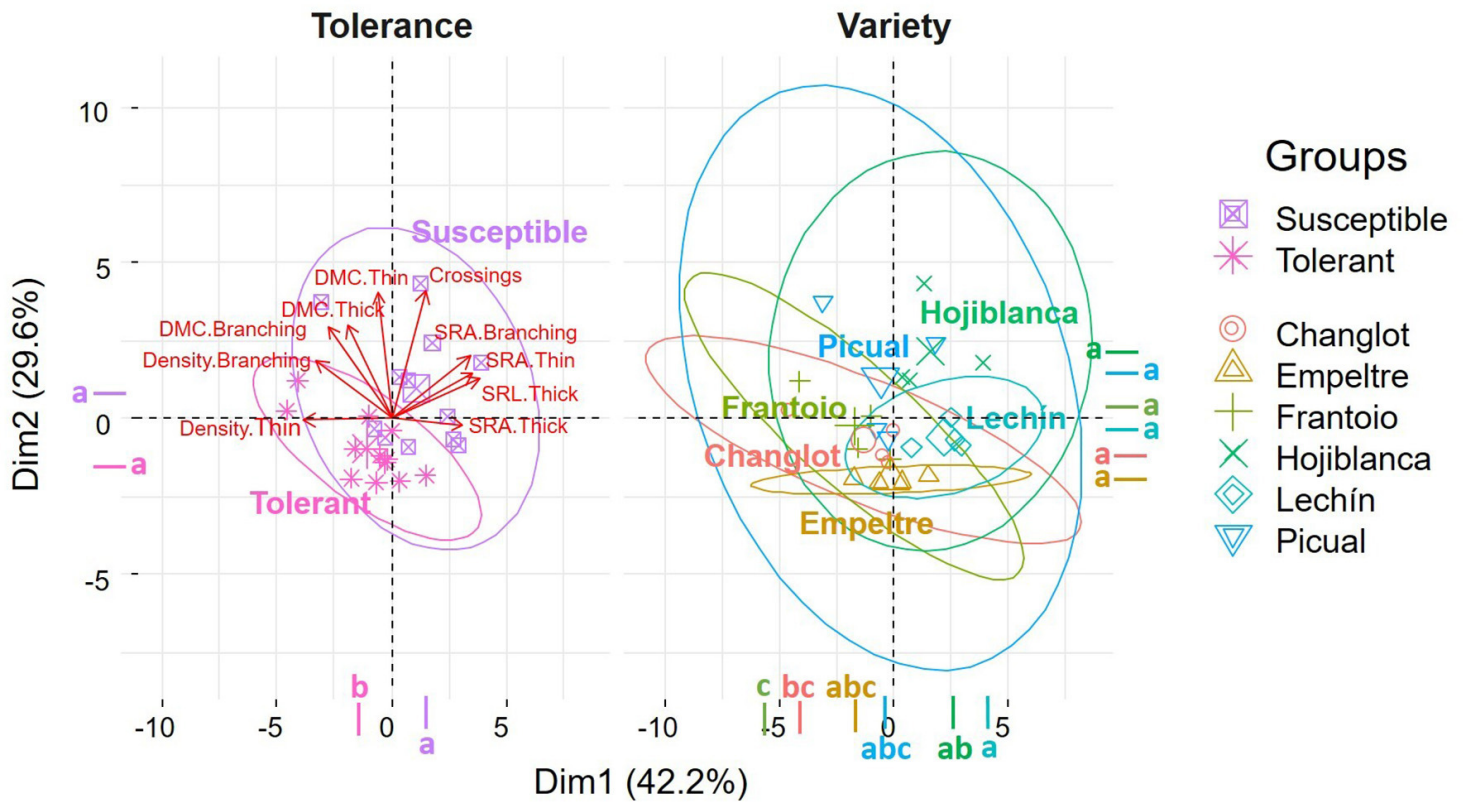
Principal Component Analysis (PCA) of root functional traits performed with “tolerance” and “variety” as factors. Different letters indicate significant differences between groups (Tukey's test, *p* < 0.05). Functional traits with a contribution to the two dimensions higher than 5% were considered for the analysis.

The PCA performed on the root system functional traits explained more than 71% of the total variance (Dim.1 = 42.2%, Dim.2 = 29.6%), with a major contribution of density thin (16.6%) and SRA thin (16.5%) for the first axis, and of crossings (26%) and DMC thin (25.4%) for the second one. This analysis separated tolerant and susceptible cultivars in two different groups, with the center of the ellipses in different quadrants, and with a major influence of thin and branching portion density for the first group, and crossings, thin, and thick SRA for the susceptible varieties (Figure 3).

### Olive Defense-Related Genes Display Different Expression Profiles Depending on Their Tolerance Level to VWO

**Figure 4:**
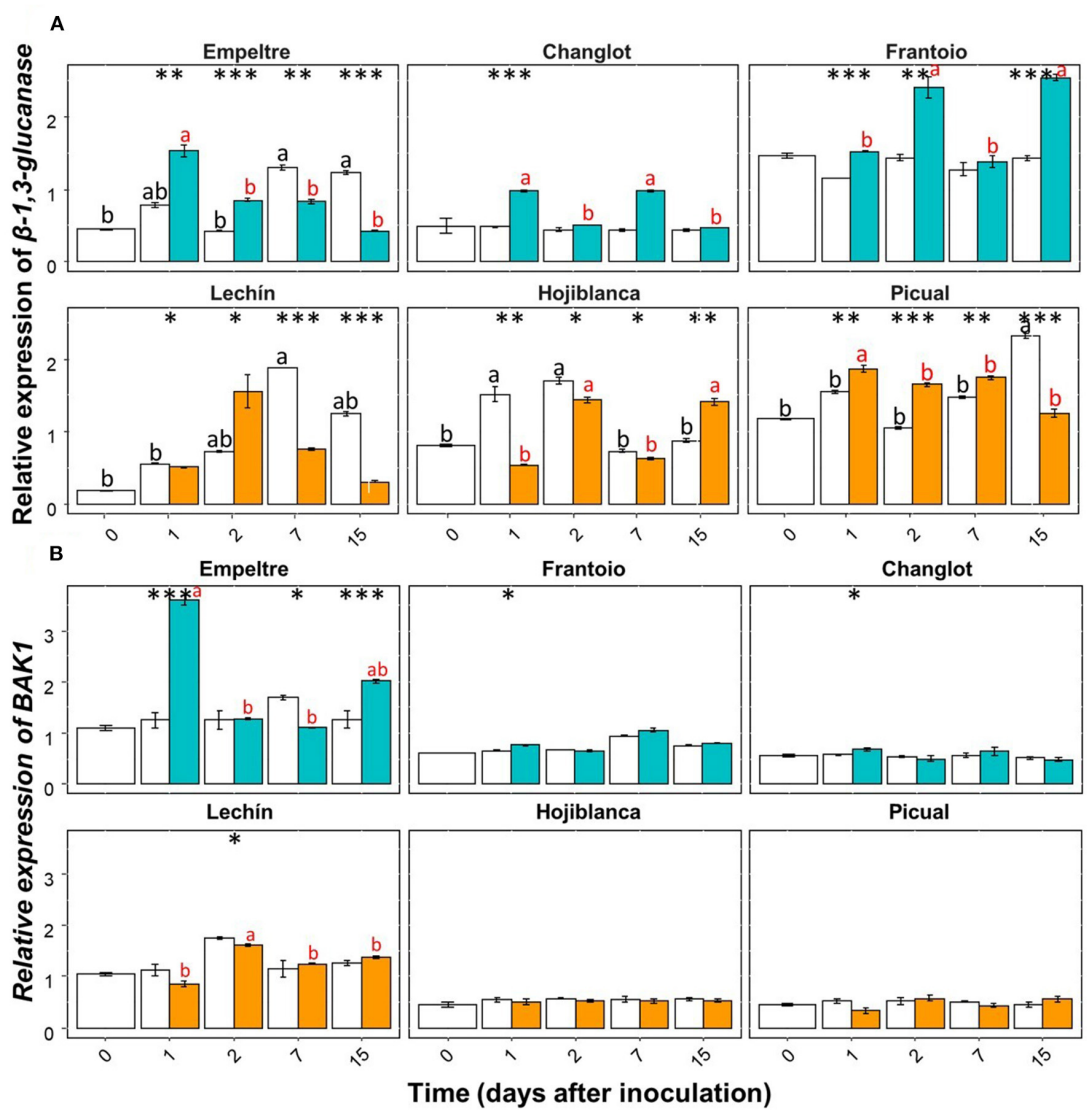
Time course of the relative expression of the β*-1,3 glucanase* **(A)**, and *BAK1* **(B)** genes. Control (non-inoculated) plants are represented in white color while *Verticillium dahliae*-inoculated plants are shown in light blue color (tolerant cultivars) or in orange color (susceptible varieties). The error bar corresponds to the standard deviation (SD) from the mean of the three biological replicates. Tukey's *post hoc* test differences (*p* < 0.05) are represented in black letters among the control plants and in red letters among inoculated plants. The statistical differences resulted by the ANOVA analysis between the control and inoculated plants are represented by asterisks (level of significance: *, *p* < 0.05; **, *p* < 0.01; ***, *p* < 0.001).

**Figure 5:**
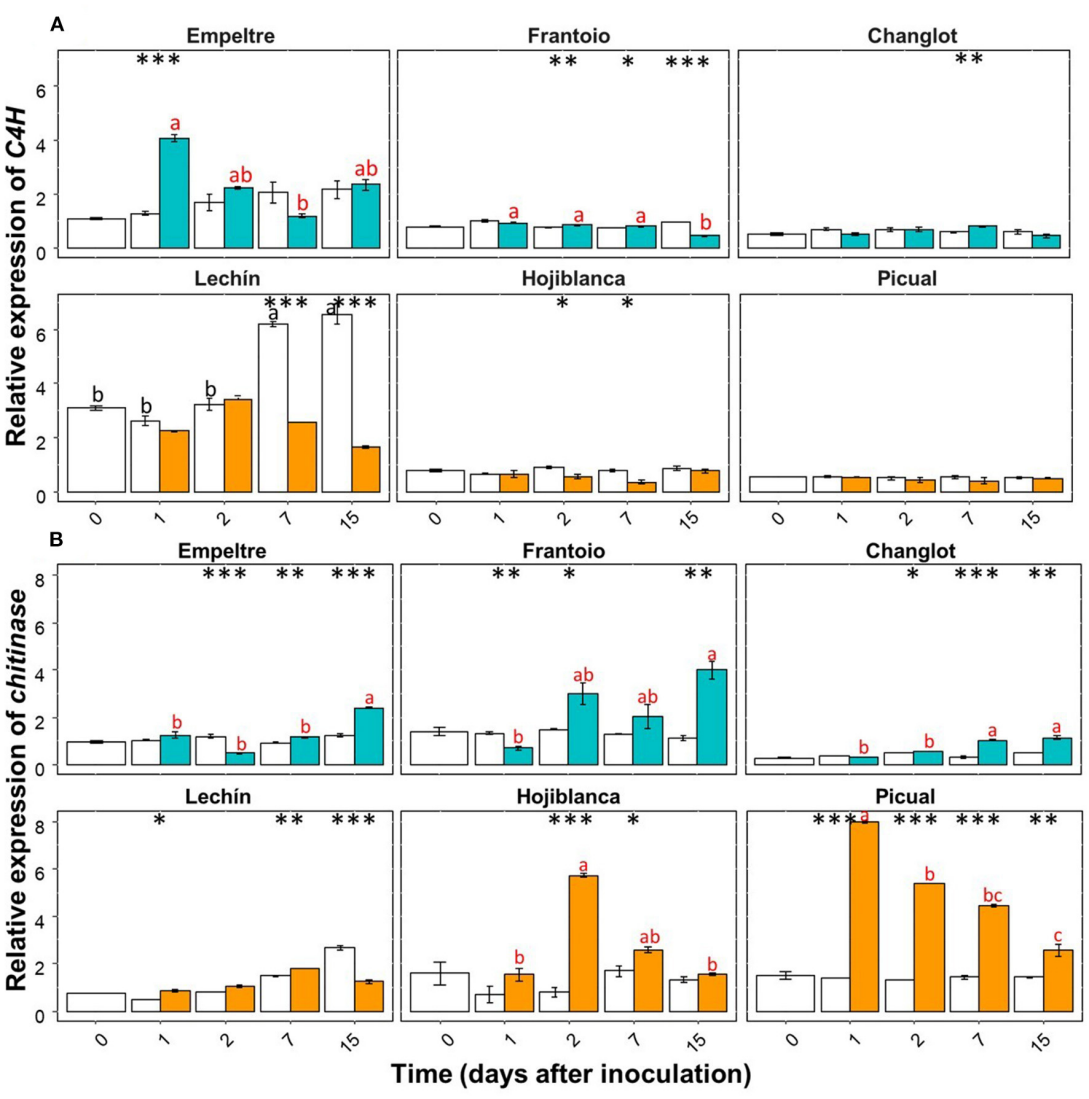
Time course of the relative expression of *C4H* **(A)** and *CO-MT* **(B)** genes. Control (non-inoculated) plants are represented in white color while *Verticillium dahliae*-inoculated plants are shown in light blue color (tolerant cultivars) or in orange color (susceptible varieties). The error bar corresponds to the standard deviation (SD) from the mean of the three biological replicates. Tukey's *post hoc* test differences (*p* < 0.05) are represented in black letters among the control plants and in red letters among the inoculated plants. The statistical differences resulted by the ANOVA analysis between the control and inoculated plants are represented by asterisks (level of significance: *, *p* < 0.05; **, *p* < 0.01; ***, *p* < 0.001).

**Figure 6:**
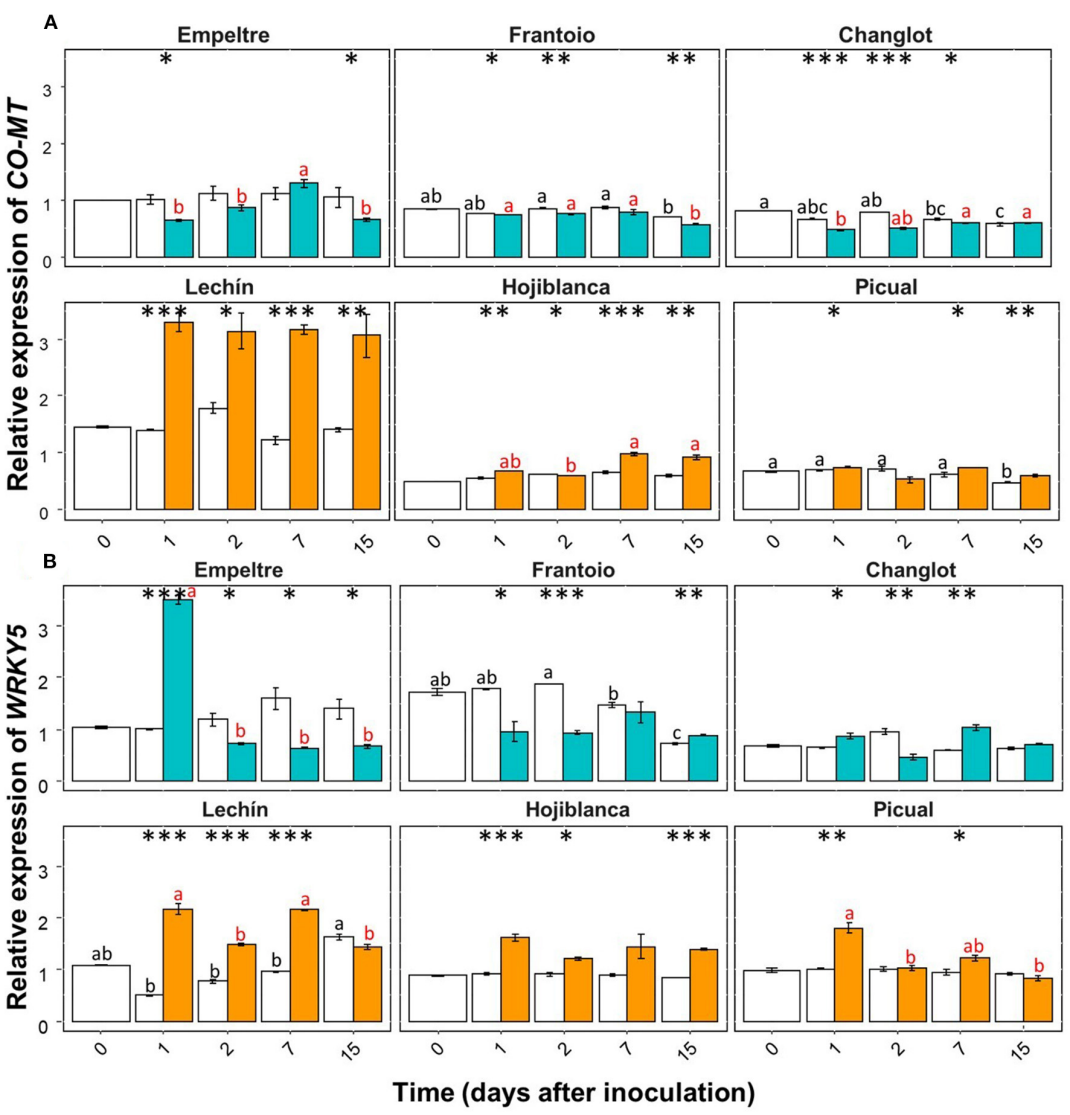
Time course of the relative expression of the *CO-MT* **(A)** and *WRKY5* **(B)** genes. Control (non-inoculated) plants are represented in white color while *Verticillium dahliae*-inoculated plants are shown in light blue color (tolerant cultivars) or in orange color (susceptible varieties). The error bar corresponds to the standard deviation (SD) from the mean of the three biological replicates. Tukey's *post hoc* test differences (*p* < 0.05) are represented with black letters among the control plants and in red letters among the inoculated plants. The statistical differences resulted by the ANOVA analysis between the control and inoculated plants are represented by asterisks (level of significance: *, *p* < 0.05; **, *p* < 0.01; ***, *p* < 0.001).

Some of the selected olive genes could be divided into two groups according to their differential expression patterns. A group of genes (e.g., β*-1,3-glucanase, BAK1, C4H, chitinase*, and *DRR2*) showed an overall significantly higher upregulation in *V. dahliae*-inoculated plants of tolerant varieties in comparison to that of the susceptible cultivars (Supplementary Figures 1, 2). In contrast, another group of genes (e.g., *CO-MT* and *WRKY5*) significantly increased their expression in the inoculated plants of the susceptible varieties (Supplementary Figure 3). Only *APX* showed an overall decreased expression in the inoculated plants of the susceptible cultivars (Supplementary Figure 1A). Furthermore, this gene showed a significantly lower expression in *V. dahliae*-inoculated plants of “Lechín de Sevilla” (*p* < 0.001) and “Picual” (*p* < 0.05) (VWO susceptible) compared to the control plants (Supplementary Figure 1A). When considering the time course, no relevant differences in *APX* gene expression were detected between the tolerant and susceptible cultivars (Supplementary Figure 4). Considering the entire time interval, the expression of the *POX* gene significantly (*p* < 0.001) decreased in the inoculated plants of “Frantoio”, whereas in *V. dahliae*-inoculated “Hojiblanca” plants, a significant (*p* < 0.05) decrease was observed (Supplementary Figure 3B). Time-course expression of this gene did not show clear differences among the olive varieties examined (Supplementary Figure 5). Regarding the first group of genes, β*-1,3-glucanase* showed a significant (*p* < 0.001) expression increase in *V. dahliae*-inoculated plants of “Changlot Real” and “Frantoio” (VWO tolerant), while no significant differences were detected in the other varieties between treatments (Supplementary Figure 1B). Considering the time course, no relevant differences between the tolerant and susceptible varieties were detected for this gene (Figure 4A). Expression of *BAK1* significantly increased only in pathogen-inoculated plants of “Empeltre” (*p* < 0.05) (Supplementary Figure 1C). Time course expression of this gene showed early (1 DAI) significant upregulation in *V. dahliae*-inoculated plants of all tolerant cultivars. In contrast, susceptible varieties remained unaltered but “Lechín de Sevilla” exhibited a significant reduction in *BAK1* expression in inoculated plants at 2 DAI (Figure 4B). Gene *C4H* showed an overall expression increase in *V. dahliae*-inoculated “Empeltre” (*p* < 0.05) but a decrease in “Hojiblanca” (*p* < 0.01) (Supplementary Figure 2A). This differential trend between the tolerant and susceptible cultivars upon pathogen inoculation was confirmed when examining the time-course expression of this gene. Indeed, tolerant varieties (“Empeltre”, “Frantoio” and “Changlot Real”) showed a significant increase in *C4H* expression between 1 and 7 DAI, whereas the susceptible cultivars (“Lechín de Sevilla” and “Hojiblanca”) displayed a decrease between 2 and 15 DAI in *V. dahliae*-inoculated plants (Figure 5A). Interestingly, the expression of the chitinase-coding gene was always significantly higher in *V. dahliae*-inoculated plants compared to noninoculated plants of all cultivars (Supplementary Figure 2B). Furthermore, the analysis of the time-course expression of this gene revealed two different trends. Indeed, a gradual increase over time was observed in *V. dahliae*-inoculated plants of tolerant varieties. However, after an initial upregulation at early times in the inoculated plants of the susceptible varieties (mostly at 1 or 2 DAI and particularly for “Picual” and “Hojiblanca”, *p* < 0.001), a steady decrease in gene expression was observed during the time interval examined (Figure 5B). Finally, the *DRR2* gene exhibited a significant expression increase in *V. dahliae*-inoculated “Changlot Real” (*p* < 0.001) and “Empeltre” (*p* < 0.05) plants (VWO tolerant) but a decrease in “Lechín de Sevilla” (*p* < 0.001) plants (VWO susceptible) (Supplementary Figure 2C). The most remarkable difference between the tolerant and susceptible cultivars concerning the expression of this gene over time was the early (1 DAI) and significantly higher expression observed in *V. dahliae*-inoculated plants of the tolerant varieties, particularly in “Empeltre” (*p* < 0.001), an outcome not observed in the susceptible cultivars (Supplementary Figure 6). Regarding the second group of genes, the expression of the gene coding for CO-MT showed a significant reduction in *V. dahliae*-inoculated plants of all tolerant varieties compared to the opposite result observed in the susceptible cultivars except “Picual” (Supplementary Figure 3A). These genes showed clear opposite expression trends from the first sampling time depending on the VWO tolerance/susceptibility level of the cultivars; that is, a decrease in the inoculated plants of tolerant cultivars and an increase in the susceptible ones (Figure 6A). Finally, gene *WRKY5* showed a significant upregulation in *V. dahliae*-inoculated plants of the susceptible cultivars, “Lechín de Sevilla” and “Hojiblanca”, an outcome not detected in tolerant plants, particularly in “Frantoio” that showed a significant expression decrease (Supplementary Figure 3C). Considering the time course, the tolerant cultivars “Empeltre” showed a decrease in *WRKY5* expression; in contrast, the susceptible varieties showed significantly higher expression in the inoculated plants compared to the control in almost all the time samplings (Figure 6B).

### Olive Cultivars Tolerant to *Verticillium dahliae* Have Higher Basal and Pathogen-Induced Root Lignin Content

**Figure 7:**
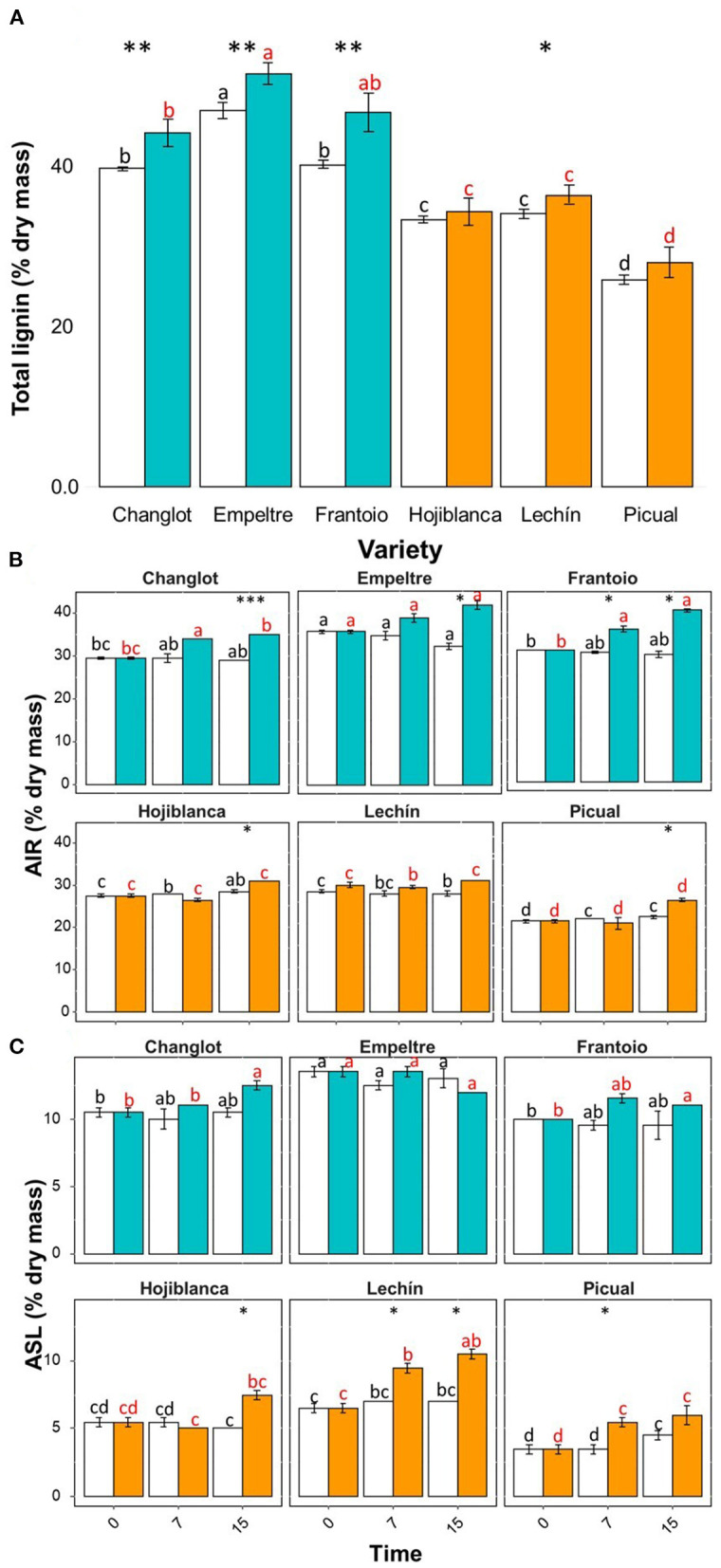
Total lignin **(A)**, acid-insoluble residue (AIR). **(B)** and acid-soluble lignin (ASL) **(C)** contents for the six olive varieties under study considering three time-points (0, 7, and 15 DAI) after inoculation with *Verticillium dahliae*. In Panel **(A)**, data are the average of nine plants (*n* = 9; three plants per time-point), while in the other two panels data are the average (*n* = 3) for each sampling time. Control (non-inoculated) plants are represented in white color and *V. dahliae*-inoculated plant in light blue for tolerant cultivars and in orange for the susceptible ones. The error bars correspond to the standard deviation (SD). The differences analyzed with Tukey's *post hoc* test (*p* < 0.05) are represented in black letters among the control plants and in red letters among the pathogen-inoculated plants. The statistical differences resulted by the ANOVA analysis between the control and inoculated plants are represented by asterisks (level of significance: *, *p* < 0.05; **, *p* < 0.01; ***, *p* < 0.001).

Lignin quantification (i.e., total lignin, AIR, and ASL) clearly showed a significantly higher basal content in the VWO tolerant cultivars. Indeed, considering the complete time interval (i.e., all plants sampled for 15 days), the tolerant varieties exhibited higher content of lignin in the roots of the control plants (Tukey *post hoc* test *p* < 0.05) compared to the susceptible ones, ‘Empeltre' showing the highest value and “Picual” the lowest one (Figure 7). Furthermore, when assessing *V. dahliae*-inoculated plants, the lignin content (Total, AIR, and ASL) was always significantly higher in the tolerant cultivars either when considering the entire interval (Figure 7A) or during the time course (Figures 7B,C). Finally, the AIR and ASL fractions showed differences along time. The AIR fraction, higher in “Empeltre” and “Frantoio” from the first sampling time compared to the susceptible cultivars (Tukey *post hoc* test *p* < 0.05) increased significantly at 15 DAI in all *V. dahliae*-inoculated plants but in “Lechín de Sevilla” (Figure 7B). On the contrary, the ASL fraction, also significantly higher in the tolerant cultivars compared to the susceptible ones (Tukey's *post hoc* test *p* < 0.05), did not show significant changes over time between the control and *V. dahliae*-inoculated plants in any tolerant cultivar, while significant increases in the inoculated plants of the susceptible varieties were found (Figure 7C).

### Root Membrane Permeability in Tolerant Cultivars Increases in the Presence of *Verticillium dahliae*

**Figure 8:**
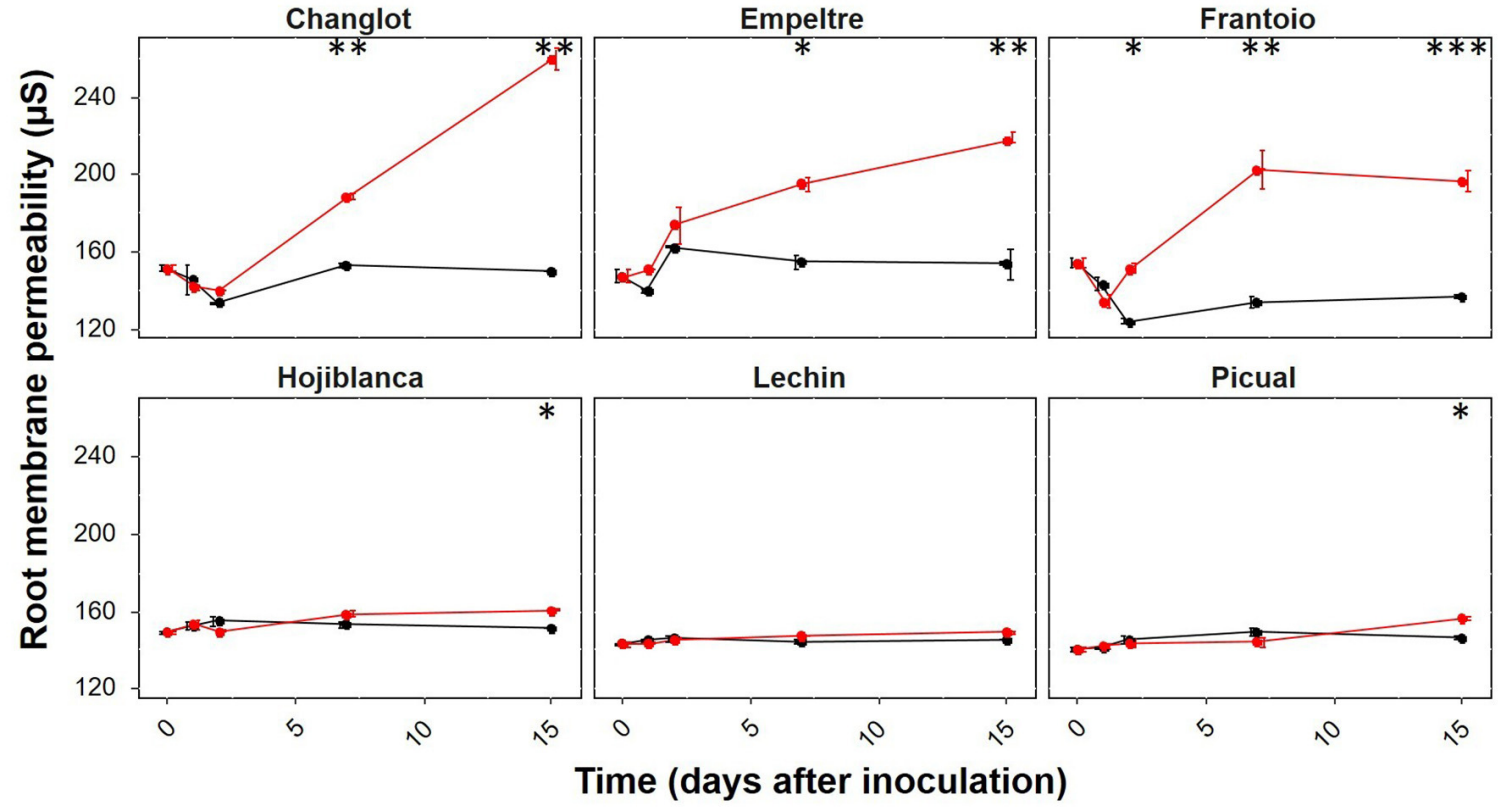
Root membrane permeability of the six olive varieties tested along time after inoculation with *Verticillium dahliae* [0, 1, 2, 7, and 15 days after inoculation (DAI)]. Data are the average (*n* = 3) for each sampling time. Non-inoculated plants are represented in black while *V. dahliae*-inoculated plants are in red. The error bars correspond to the standard deviation (SD) from the mean of the three replicates. Statistical differences at each time-point (ANOVA) between the control and inoculated plants are represented by asterisks (level of significance: *, *p* < 0.05; **, *p* < 0.01; ***, *p* < 0.001).

Before pathogen inoculation, differences in membrane permeability among the tested cultivars were not significant (*p* > 0.05). After inoculation with *V. dahliae*, the membrane permeability significantly increased in all olive varieties under study but “Lechín de Sevilla”. Remarkably, root membrane permeability was sharply augmented in all tolerant cultivars, particularly at 7 DAI, compared to the null or moderate increase observed in susceptible varieties. Nonetheless, “Picual” and “Hojiblanca” also showed a significant increase in *V. dahliae*-inoculated plants at 15 DAI (*p* < 0.05), although increments were nearly negligible compared to those scored for tolerant cultivars (Figure 8).

## Discussion

The olive cultivars here under study showed significant differences in several functional root traits, and some of them could be linked to their distinctive level of tolerance/susceptibility to VWO. Therefore, the root system architecture contributed, at least to some extent, to explain olive cultivar performance against *V. dahliae*. Susceptible varieties showed higher SRL and SRA values for all root classes (thin, thick, and branching portions) but lower AvgDiam values for thin roots. The SRL trait determines how much root length is built per unit of the root mass (Ostonen et al., 2007). Thus, the increase of SRL enhances the root–soil interface (Ryser, 2006). Usually, variations in SRL are proportional to those of SRA (Lõhmus et al., 1989; de la Riva et al., 2018), which represents the root surface area per root mass, as found in this work. Besides, roots with high SRL present a small average diameter (Eissenstat, 1992), another structural correlation confirmed by our results. Furthermore, roots with high SRL and small diameter are more plastic in lateral root proliferation, obtaining greater root length densities than species with lower SRL (Mengel and Steffens, 1985; Eissenstat, 1992; de la Riva et al., 2021). These results confirmed our earlier finding that VWO-susceptible cultivars presented more branched root systems with more plasticity and more thin roots (Cardoni et al., 2021). Moreover, in this present work, we found that VWO-susceptible varieties presented a higher number of tips, forks, and crossings compared to the tolerant ones. Root tips are the points where root elongation takes place through the generation of new cells from meristematic tissue within the apical region (Curlango-Rivera and Hawes, 2011). The larger number of tips and the higher SRL values found in VWO-susceptible plants may indicate that these cultivars invest more resources in growth and development processes compared to the tolerant ones (Ostonen et al., 2007). Interestingly enough, this is in accordance with the recent results indicating that gene expressions in the roots of VWO-susceptible cultivars were more devoted to plant growth than that of tolerant varieties, which in contrast invested more in processes, such as defense against pathogens (Ramírez-Tejero et al., 2021). Furthermore, susceptible cultivars also showed larger number of forks, i.e., root ramification points, and crossings, i.e., root intersections, compared to the tolerant varieties, confirming the presence of more laterally developed, plastic, and branched root systems in VWO-susceptible varieties. Higher number of tips and forks would mean that roots are more exposed to pathogen attacks. Indeed, newly generated tissue is highly susceptible to infection by pathogens. In legumes, for example, more than 90% of bulk carbon released from the young roots is delivered by the root cap, a small zone localized in the root apex (Griffin et al., 1976). It has been reported that some pathogens are attracted specifically to the root tip region presumably in response to such exudates (Curlango-Rivera and Hawes, 2011). For instance, Goldberg et al. (1989) demonstrated that *Pythium dissotocum* was attracted by cotton roots placed in a deposition of pathogen zoospores, and that this host specific attraction took place on the root tip region where border cells were present.

Roots of VWO-susceptible olive varieties can also be more prone to be invaded by soil-borne pathogens due to their significantly lower basal content of lignin (AIR, ASL, and Total) compared to that of tolerant cultivars, as reported here. Furthermore, tolerant cultivars showed higher increases in the total lignin content of the roots, linked to the increase of the AIR fraction after *V. dahliae* inoculation, compared to the susceptible varieties that did not display such increases (or were almost null). Similar results were found in *V. dahliae*-tolerant cotton hypocotyls (Smit and Dubery, 1997) and in the roots of *V. dahliae*-tolerant tomato plants (Gayoso et al., 2010) infected by the pathogen, that showed faster and more intense synthesis and deposition of lignin compared to the susceptible ones. Evidence of higher velocity to synthesize lignin polymers in tolerant plants compared to susceptible ones after pathogen inoculation were also found in olive roots inoculated with *V. dahliae* (Gharbi et al., 2017b) and in olive leaves inoculated with *X. fastidiosa* (Sabella et al., 2018). The deposition of lignin in the plant cell wall not only provides a mechanical barrier against pathogen penetration but also provides protection to cell wall polysaccharides from being degraded by enzymes released by the invader (Gharbi et al., 2017b). The important role of olive cell walls lignification to hinder pathogen attacks has already been described (Smit and Dubery, 1997; Gayoso et al., 2010; Njoroge et al., 2011; Xu et al., 2011; Gharbi et al., 2017b; Sabella et al., 2018; Cardoni et al., 2021). It is plausible to think that an olive root system presenting more thin roots, more branches, and low lignin content, characteristics predominantly found in susceptible cultivars, is not able to confront the pathogen as efficiently as the more lignified, less branched, and thicker roots frequently present in the tolerant cultivars. Therefore, it can be concluded that differences in the root system architecture, especially at the branching structure, and in the lignin content contribute to the performance of olive against colonization and invasion by *V. dahliae*. Interestingly, the expression patterns of the two genes involved in the lignin biosynthetic pathway evaluated in this study correlated with the results obtained from lignin content analysis. The gene *C4H*, coding for the enzyme that catalyzes the conversion of trans-cinnamic acid to p-coumaric acid, the first hydroxylation step in the lignin biosynthetic pathway (Yan et al., 2019), increased its expression in *V. dahliae*-inoculated plants of tolerant cultivars. The importance of this gene in resistance to pathogens has been described in soybean. Indeed, the tissue-specific silencing of *GmC4H1* in the hairy roots of soybean led to reduced resistance against the oomycete, *Phytophthora sojae* (Yan et al., 2019). Remarkably, the expression trend displayed by this gene correlated with the increase in total lignin and AIR content observed in VWO-tolerant cultivars, while no significant variation was found in VWO-susceptible ones. On the contrary, the *CO-MT* gene, involved in the synthesis of sinapyl alcohol (S) monolignol, one of the three monolignoles that compose the lignin polymer (Peter and Neale, 2004), showed an increase in the susceptible cultivars and a decrease in the tolerant ones. Similar results were earlier found in the stems of “Frantoio” between 4 and 10 DAI with *V. dahliae* (Gómez-Lama Cabanás et al., 2015). Concomitant with the *CO-MT* expression pattern, susceptible cultivars also showed an increase in the ASL content, which was not detected in the tolerant ones that showed a higher content of ASL from the beginning of the experiment. The ASL fraction is strongly related to the S monolignol content, due to the higher reactivity of this monomer to sulfuric acid compared to the guaiacyl (G) monolignol, the other most abundant monomer present in lignins of angiosperm dicots, which are easier to be detected by the AIR index (Yasuda et al., 2001; Vanholme et al., 2010). An increase in the G/S monolignols ratio was also reported in the tomato roots inoculated with *V. dahliae*, but without significant differences between tolerant and susceptible varieties (Gayoso et al., 2010). Moreover, an increased expression was also found for *WRKY5* in the VWO-susceptible varieties. Hu et al. (2021) reported a link between the expression of another gene-coding transcriptional factor of the WRKY family, *WRKY1*, with that of *CO-MT1* in cotton roots inoculated with *V. dahliae*. In the study by Hu et al., the overexpression of *WRKY1* was related to the direct activation of genes involved in lignin synthesis, such as *CO-MT1*, and with a significant accumulation of S monolignol content. An increase in S monolignin was also found in the leaves of wheat plants inoculated with the pathogenic fungus, *Puccina graminis* during the hypersensitive response upon fungal penetration (Menden et al., 2007). It is tempting to speculate that a higher initial amount of lignin (both ASL and AIR fractions) and an increase in AIR lignin after pathogen inoculation could be related with an enhanced tolerance of olive plants to *V. dahliae*. On the contrary, the late rise of S monolignol accumulation (ASL) and the lack in G monolignol production (AIR), confirmed by the increase in *WRKY5* and *CO-MT* and the decrease of *C4H* expression in susceptible cultivars respectively, could be related to higher susceptibility to this pathogen. More studies are needed in-depth to analyze the changes in the G/S ratio and in the lignin monomers conformation after *V. dahliae* inoculation and their relationship with pathogen tolerance in olive roots.

Differences between VWO-tolerant and VWO-susceptible olive cultivars related to PR proteins-based defense mechanisms were also examined in this study. Genes coding for chitinase and β-1,3-glucanase significantly increased their expression in *V. dahliae*-inoculated plants of the tolerant varieties, as reported elsewhere with different olive cultivars (Gharbi et al., 2017b). The similar expression trend found for these two genes confirmed the hypothesis of Balasubramanian et al. (2012); that is, both genes work coordinately to degrade the fungal cell wall. The important role that these enzymes play in plant defense against *V. dahliae* has been extensively described in cotton (Wu et al., 2004; Hongmei C. G. N. W., 2005) and tomato (Young and Pegg, 1982). Interestingly, after the rapid induction of the chitinase-coding gene observed in all cultivars immediately after *V. dahliae* inoculation, a gradual decrease in its expression followed only in the susceptible plants. It is tempting to speculate that this finding could be related to the inability of these cultivars to slow down the root colonization process by the pathogen.

The ability to activate innate immunity seemed to be also different between susceptible and tolerant cultivars. Indeed, *BAK1* is a positive regulator of the PAMPs receptor that plays a functional role in pattern recognition and subsequent activation of innate immunity (Chinchilla et al., 2007). Upregulation of *BAK1* has been reported to be required for Verticillium wilt resistance in *A. thaliana* (Chinchilla et al., 2007), tomato (Fradin et al., 2009), cotton (Gao et al., 2013), and olive (Leyva-Pérez et al., 2018) plants. Our results are in agreement with the above studies since pathogen-inoculated plants of tolerant varieties showed a significant increase in *BAK1* expression. The gene *DRR2* showed an increased expression in the inoculated plants of the tolerant cultivars, although no clear correlation was found between its expression pattern and the susceptibility/tolerance level to VWO.

Finally, regarding genes related to ROS response (*APX* and *POX*), no clear differences during the time course analysis were detected in this study. Therefore, the results obtained did not allow for establishing a clear correlation between tolerance/susceptibility of the six cultivars to *V. dahliae* examined and the expression of these genes.

Relevant differences between *V. dahliae*-tolerant and susceptible cultivars were also observed at the plant cell membrane level. Indeed, the significant rise found in the root membrane permeability of the tolerant cultivars upon pathogen inoculation confirmed that this phenomenon is associated with disease resistance as earlier reported in other studies carried out in black pepper infected by *Phytophthora capsici* (Vandana et al., 2014) and olive inoculated with *V. dahliae* (Gharbi et al., 2017b). In fact, other studies showed that the increase in membrane permeability is the earliest reaction of the plant cell to fungal elicitors (Cheng et al., 2013; Gharbi et al., 2017b). Variations in membrane permeability were related to calcium and proton influx and potassium and chloride efflux which are essential for oxidative burst initiation, defense genes induction, and metabolites production (Wan et al., 2008). The increase in membrane permeability was also related to improved integrity and stability of the plasma membrane of root cells due to increasing P uptake. Indeed, mycorrhizal roots of maize plants under salt stress presented higher membrane permeability compared to the roots noncolonized by the symbiotic fungus (Feng et al., 2002). A rapid increase of membrane permeability in tolerant cultivars could indicate a faster reaction of these plants to the presence of the pathogen compared with the susceptible ones. So far, this is the first work that shows the existence of a link between variations in membrane permeability and pathogen tolerance at the subspecies level (i.e., cultivars).

Interesting relationships between physiological, morphological, and genetics parameters here analyzed and VWO development and severity could be observed for each variety. Regarding susceptible cultivars, “Picual” showed the highest disease parameters in our bioassay and from the traits examined, it can be asserted that their high susceptibility is determined, at least to some extent, by the morphology and specific physiological responses of its root system. Indeed, this cultivar showed the lowest basal values of lignin content (total, AIR, and ASL), the thinnest roots (AvgDim), and the highest number of tips. “Hojiblanca” presented the second-highest rate of plant mortality. This cultivar showed the highest numbers of branching, forks, and crossings, and also the highest relative area and length of the branching. However, in contrast to “Picual”, “Hojiblanca” showed higher basal content of AIR and ASL and significantly higher total lignin content than the former. Considering the genetic responses, these two cultivars showed a rapid and significant decrease of the *chitinase* gene, unlike “Lechín de Sevilla”. Both “Picual,” and “Hojiblanca” displayed very low or null expression of *BAK1*, related to the activation of innate immunity, and *DRR2*, related to the production of a disease resistance-responsive protein. “Lechín de Sevilla” showed the second-highest disease incidence but the lowest percentage of dead plants among the evaluated susceptible cultivars. Considering the morphological root traits of this cultivar, no significant differences were found for the number of tips, crossings, and forks compared to the tolerant varieties. However, this cultivar showed the highest specific length and area of thin roots. Furthermore, regarding lignin, “Lechín de Sevilla” showed the highest basal ASL content among the susceptible varieties and a significant increase of total lignin in *V. dahliae*-inoculated plants compared to the control ones. It also showed a significant increase in the expression of *CO-MT* and *WRKY5* genes, which are related to the increase of the ASL fraction. Unlike the other two susceptible cultivars, “Lechín de Sevilla” plants (both control and inoculated) showed higher expression of the *BAK1* and *DRR2* genes and lower decrease over time of *chitinase* gene expression. The three susceptible cultivars showed lower values of membrane permeability in pathogen-inoculated plants compared to the tolerant ones. Moreover, this parameter remained nearly unaltered at the end of the experiment in VWO-susceptible plants. Regarding the tolerant cultivars, the disease did not develop in our experiment. This outcome could be due, among other factors, to their high basal lignin content and by the increase of the AIR fraction (and no changes in ASL content) upon *V. dahliae* inoculation. This is supported by the observed increase in *C4H* (and decrease of *CO-MT* and *WRKY5*) gene expression in all these varieties after inoculation with the pathogen. This group of cultivars always showed similar trends in the expression of the genes evaluated, unlike the susceptible varieties that displayed more variability. Regarding the morphological parameters “Empeltre” showed the clearest differences (i.e., the lowest number of forks, crossings and tips, and specific area and length of branching) compared to the susceptible cultivars. Furthermore, all tolerant cultivars showed higher values of thin root diameter and lower values of thin root-specific area. Finally, the increase in root membrane permeability in *V. dahliae*-inoculated plants of these cultivars displayed a similar trend, with “Changlot Real” plants showing the highest value at the end of the experiment.

**Figure 9:**
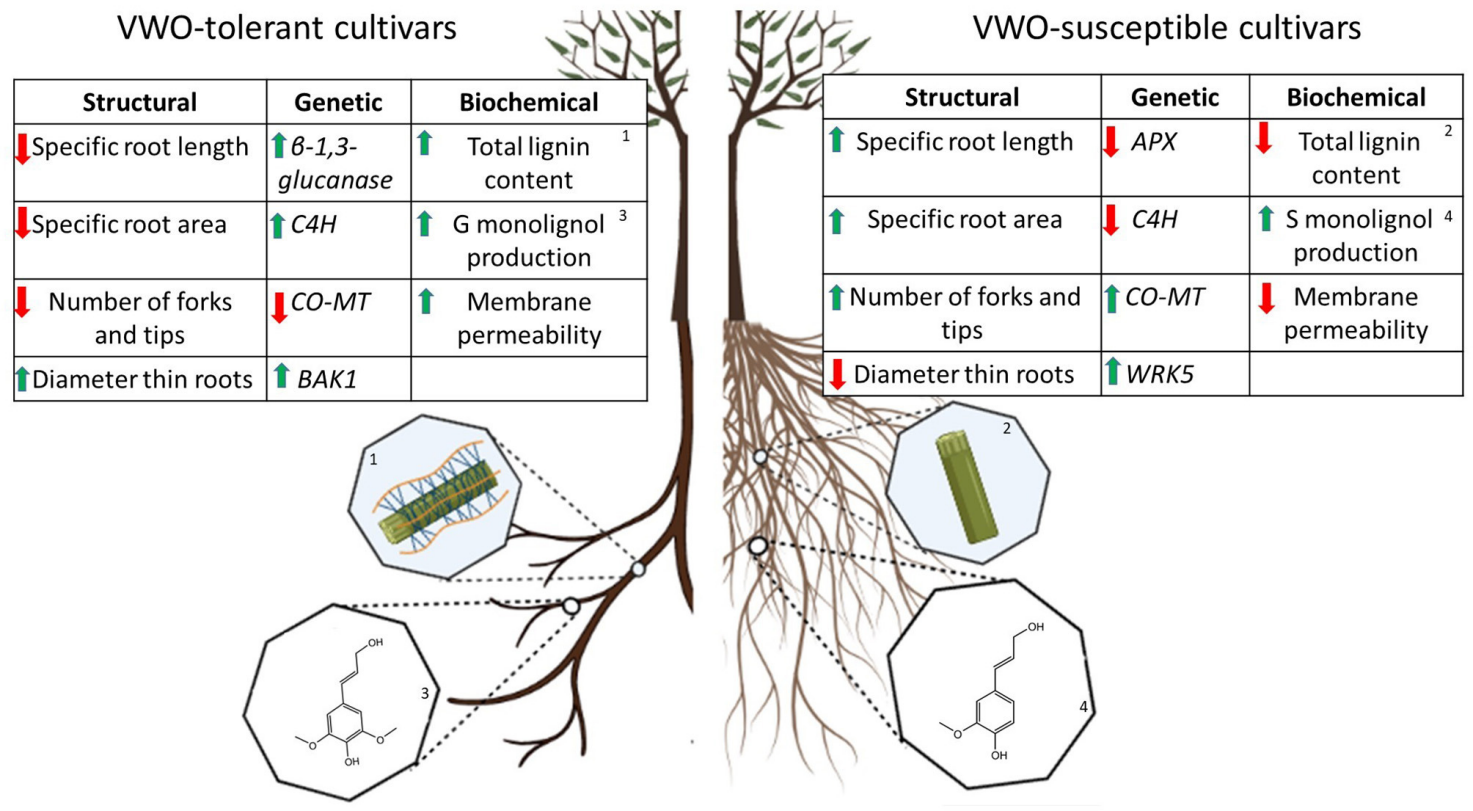
Schematic summary of the major differences related to defense mechanisms found between Verticillium wilt of olive (VWO)-tolerant (**left**) and VWO-susceptible (**right**) olive cultivars at the root level. In the tables, the first column shows the most significant differences found in the root system architecture, in the absence of the pathogen. The second and third columns report the differences observed at the genetic and biochemical levels, respectively, after *Verticillium dahliae* inoculation. Increases are shown by green arrows, while decreases are represented by red arrows. The ^1^high or ^2^low content of lignin in the roots are represented by the major quantity or absence of the lignin polymers (blue lines) in the lignocellulose structure (green rectangle), respectively. ^3^High G monolignol content in the roots is represented by the chemical structure of coniferyl alcohol, while ^4^high S monolignol presence is represented by the chemical structure of sinapyl (S) alcohol.

The most important findings of this study are summarized in Figure 9. First of all, the root system architecture contributed to the ability of a given olive cultivar to cope with eventual attacks by *V. dahliae*. Indeed, the large root volume and a high number of lateral branching and forks found in the VWO-susceptible varieties seem to explain their proneness to be infected by propagules of the pathogen present in the soil. Therefore, the first hypothesis to be tested in this study has been confirmed: there is a relationship between the branching root architecture, mostly related to the number of forks, tips, and crossings, and the tolerance/susceptibility of olive cultivars to *V. dahliae*. Second, the lower basal lignin content present in the roots of the susceptible plants compared to that found in the tolerant cultivars represented another weakness of the former varieties to effectively confront the pathogen. This is confirmed in our previous studies about the important role of olive root lignin content in tolerance against *V. dahliae*. Third, from the genetic point of view tolerant cultivars seemed to be more prepared to effectively respond to colonization and invasion by the pathogen. Indeed, VWO-tolerant varieties activated genes related to defense mechanisms from the early days of *V. dahliae* inoculation, in contrast to the susceptible ones. These genes are involved in cell wall lignin biosynthesis (i.e., *C4H* and *CO-MT*), as confirmed by the biochemical analysis of the pathogen-induced root lignin content, in the production of hydrolytic enzymes which can degrade the fungal cell wall (i.e., β*-1.3-glucanase*) and in the activation of innate immunity (i.e., *BAK1* and *WRKY5)*. In a similar scenario, the susceptible cultivars showed a delay or lack of expression of these genes. Consequently, the second hypothesis to be tested in this work has been also confirmed: the VWO tolerance level of olive cultivars can be explained by the different timings and intensity of genetic defense responses. This study is the first to elucidate the relationship between olive root architecture and the biochemical composition and VWO resistance, from both genetic and functional points of view, considering the presence and absence of the pathogen. However, more studies are still needed to fill the gaps of the complex, multilayered defense mechanism underlying the interaction between olive and *V. dahliae*. Studies integrating multidisciplinary approaches, including genomics, metabolomics, and plant physiology, are encouraged to comprehensively understand the mechanisms of resistance/tolerance to *V. dahliae*, thereby contributing to generate innovative and more holistic alternatives for the effective management of VWO as well as novel tools in breeding resistance to this disease.

## Data Availability Statement

The original contributions presented in the study are included in the article/Supplementary Material, further inquiries can be directed to the corresponding author.

## Author Contributions

JM-B conceived the study. MC, CG-L, and JM-B designed the experiments. MC and AV-C carried out samplings. MC performed all experiments. CG-L contributed to the execution of genetic experiments. RV contributed to the functional traits analysis. MC and JM-B wrote the article. All authors have read and agreed to the published version of the manuscript.

## Funding

This work was supported by the Spanish Ministerio de Ciencia e Innovación/Agencia Estatal de Investigación (grant PID2019-106283RB-I00) and projects UCO-FEDER 18 REF 27943 MOD B and P18-RT-3455 from Junta de Andalucía (Spain). All co-funded with EU FEDER funds.

## Conflict of Interest

The authors declare that the research was conducted in the absence of any commercial or financial relationships that could be construed as a potential conflict of interest.

## Publisher's Note

All claims expressed in this article are solely those of the authors and do not necessarily represent those of their affiliated organizations, or those of the publisher, the editors and the reviewers. Any product that may be evaluated in this article, or claim that may be made by its manufacturer, is not guaranteed or endorsed by the publisher.
