# Supplementary material for: Unveiling Differences in Root Defense Mechanisms Between Tolerant and Susceptible Olive Cultivars to Verticillium dahliae
Source: Front Plant Sci. 2022 Apr 25;13:863055. doi: 10.3389/fpls.2022.863055 (PMC9083224; doi:10.3389/fpls.2022.863055)
Supplement: Supplementary file 1 [file Data_Sheet_1.docx]

Supplementary Material

Supplementary Table 1 Genes, primer pairs and RT-qPCR parameters

| **Gene Coding Function** | **Abbreviation/name** | **Reference** | **Biological Process** | **Primer sequences** | **Aplicon size** | **Annealing temperature** | **Amplification efficiency (E)** | **Correlation coefficient (r)** | **Linear equation** |
| --- | --- | --- | --- | --- | --- | --- | --- | --- | --- |
| Elongation factor 1α | *EF1-α* | Ray and Johnson (2014) | housekeeping | **F:**  GAATGGTGATGCTGGTTTCG  **R:** CCCTTCTTGGCAGCAGACTTG | 191 | 57 | 95.5% | 0.995 | y = 3.434x+21.803 |
| 60S ribosomal protein L18-3 | *60S RBP L18-3* | Ray and Johnson (2014) | housekeeping | **F:** GTAAGAGCAAGAAGACCAAG  **R:** GTAAGAGCAAGAAGACCAAG | 101 | 55 | 100.5% | 0.995 | y = 3.309x+21.562 |
| ascorbate peroxidase | *APX* | Gharbi et al., (2017a) | ROS defense | **F:** ACGAATATCGAAGGCCAGTG  **R:** GATGGGGCAAATGCTTCTTA | 250 | 60 | 101.9% | 0.95 | y = 3.278x+26.179 |
| Beta-1,3-glucanase | *β-1,3-glucanase* | Gharbi et al., (2017b) | plant defense (pathogen cell wall degradation) | **F:** TGATGGAACACGGTACCAGA  **R:**  AGCCTTTTCAAGTGCTGCAT | 70 | 60 | 106% | 0.991 | y = 3.185x+22.320 |
| cinnamate-4-hydroxylase | *C4H* | Sabella et al., (2018) | lignin pathway | **F:** CGGCATTACTTTGGGACGTTT  **R:** GCAGACTGAATTGGCCACCT | 226 | 60 | 100.7% | 0.998 | y = 3.306x+19.410 |
| chitinase | *chitinase* | Gharbi et al., (2017b) | plant defense (pathogen cell wall degradation) | **F:**  GTGCCTGGTTATGGTGTCGT  **R:**  TTCGAACCTTTACCGCATTC | 65 | 60 | 102.9% | 0.995 | y = 3.254x+20.348 |
| caffeoyl-o-methyltransferase | *CO-MT* | Schilirò et al., (2012) | lignin pathway | **F:** ACCAGAGGCCATGAAAGAAC  **R:**  ATTGCCAAAATCTTCCCATC | 204 | 55 | 93.6% | 0.997 | y = 3.485x+19.793 |
| disease-resistance-responsive protein | *DRR2* | Gómez-Lama Cabanás et al., (2015) | plant defense response | **F:**  CCAATGCCCGTAAAGTAA  **R:**  TACAGCGTTTCTTCCCAA | 311 | 55 | 100.8% | 0.998 | y = 3.303x+19.152 |
| Brassinosteroid insensitive 1-associated receptor kinase 1 | *BAK1* | Jiménez‐Ruiz et al., (2017) | plant immunity system | **F:**  GCCCTCTGCCCTGTTATTA  **R:**  GGCTGTTGTTGGGGATTT | 186 | 57 | 99.6% | 0.992 | y = 3.322x+23.564 |
| peroxidase | *POX* | Gharbi et al., (2017b) | plant defense against fungi | **F:** ACGAATATCGAAGGCCAGTG  **R:**  ATGGGGCAAATGCTTCTTA | 250 | 60 | 104.3% | 0.991 | y = 3.222x+26.306 |
| WRKY5 Transcription Factor | *WRKY 5* | Schilirò et al., (2012) | global regulators of host responses (SA signal transduction) | **F:** GCATGGTGCAAGAAGTAGGA  **R:** CAGCAACAAACGCTACACCT | 213 | 55 | 103.2% | 0.997 | y = 3.248x+21.748 |

**F,** Forward primer; **R**, reverse primer


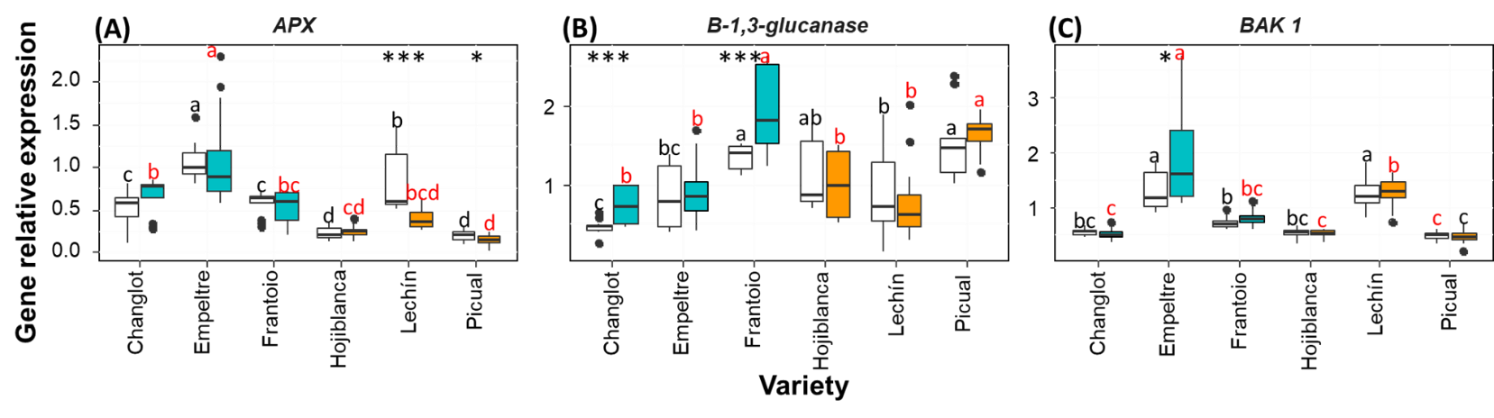


**Supplementary Figure 1** Box plots showing median values of relative expression of *APX* (A), *β-1,3-glucanase* (B) and *BAK1* (C) genes. Data are the average values of the relative gene expression in all plants (n= 15) sampled along the experiment (i.e. from 0 to 15 days after inoculation). Control (non-inoculated) plants are represented in white color and *Verticillium dahliae*-inoculated plants in light blue for tolerant cultivars and in orange for the susceptible ones. Tukey *post hoc* test differences (*p* <0.05) are represented with black letters for control plants and in red letters for inoculated plants. Statistical differences resulted by the ANOVA analysis between control and inoculated plants of each cultivar are indicated by asterisks (level of significance: *, *p* < 0.05; **, *p* < 0.01; ***, *p* < 0.001).


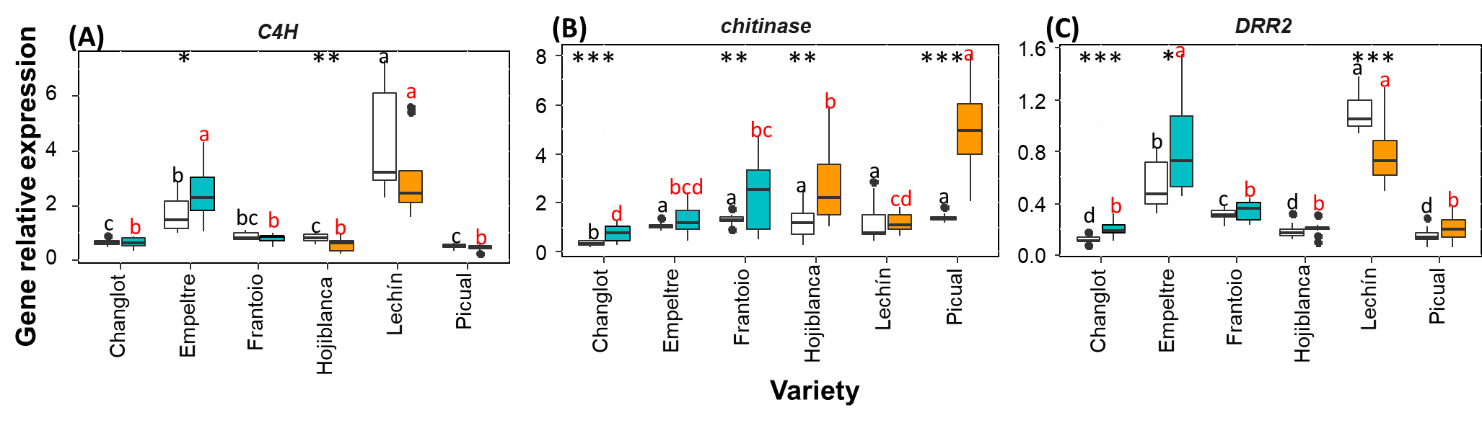


**Supplementary Figure 2** Box plots showing median values of relative expression of *C4H* (A), *chitinase* (B), and *DRR2* (C) genes. Data are the average values of the relative gene expression in all plants (n= 15) sampled along the experiment (i.e. from 0 to 15 days after inoculation). Control (non-inoculated) plants are represented in white color and *Verticillium dahliae*-inoculated plants in light blue for tolerant cultivars and in orange for the susceptible ones. Tukey *post hoc* test differences (*p* <0.05) are represented with black letters for control plants and in red letters for inoculated plants. Statistical differences resulted by the ANOVA analysis between control and inoculated plants of each cultivar are indicated by asterisks (level of significance: *, *p* < 0.05; **, *p* < 0.01; ***, *p* < 0.001).


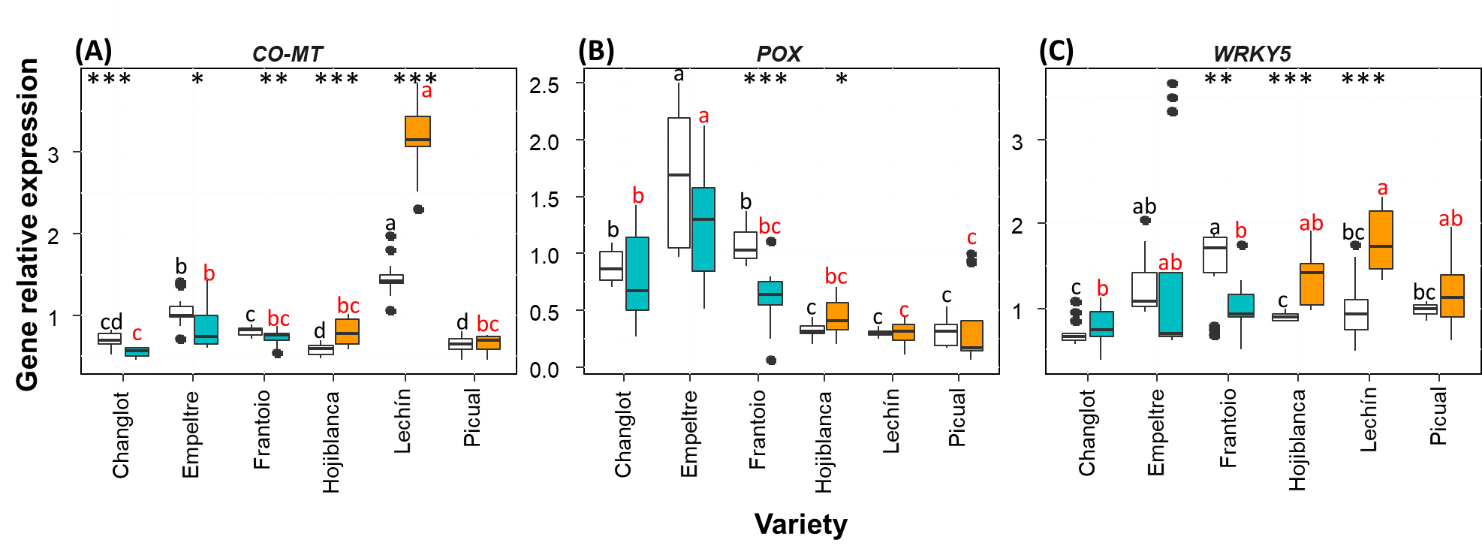


**Supplementary Figure 3** Box plots showing median values of relative expression of *CO-MT* (A), *POX* (B), and *WRKY5* (C) genes. Data are the average values of the relative gene expression in all plants (n= 15) sampled along the experiment (i.e. from 0 to 15 days after inoculation). Control (non-inoculated) plants are represented in white color and *Verticillium dahliae*-inoculated plants in light blue for tolerant cultivars and in orange for the susceptible ones. Tukey *post hoc* test differences (*p* <0.05) are represented with black letters for control plants and in red letters for inoculated plants. Statistical differences resulted by the ANOVA analysis between control and inoculated plants of each cultivar are indicated by asterisks (level of significance: *, *p* < 0.05; **, *p* < 0.01; ***, *p* < 0.001).


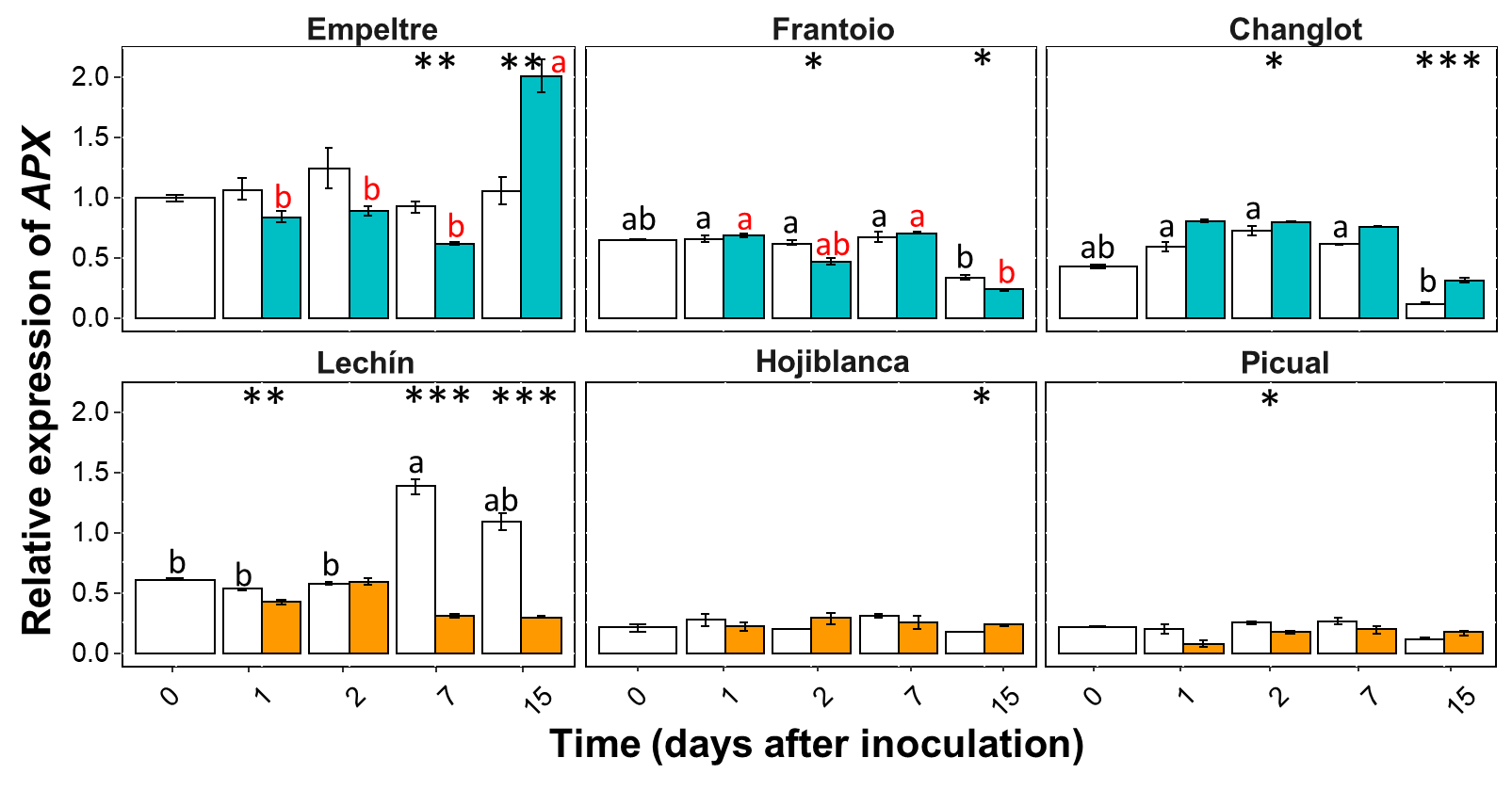


**Supplementary Figure 4** Time course of the relative expression of the *APX* gene. Control (non inoculated) plants are represented in white color while *Verticillium dahlia*e-inoculated plants are shown in light blue color (tolerant cultivars) or in orange color (susceptible varieties). The error bar corresponds to the standard deviation (SD) from the mean of the three biological replicates. Tukey *post hoc* test differences (*p* <0.05) are represented with black letters among control plants and in red letters among inoculated plants. The statistical differences resulted by the ANOVA analysis between control and inoculated plants are represented by asterisks (level of significance: *, *p* < 0.05; **, *p* < 0.01; ***, *p* < 0.001).


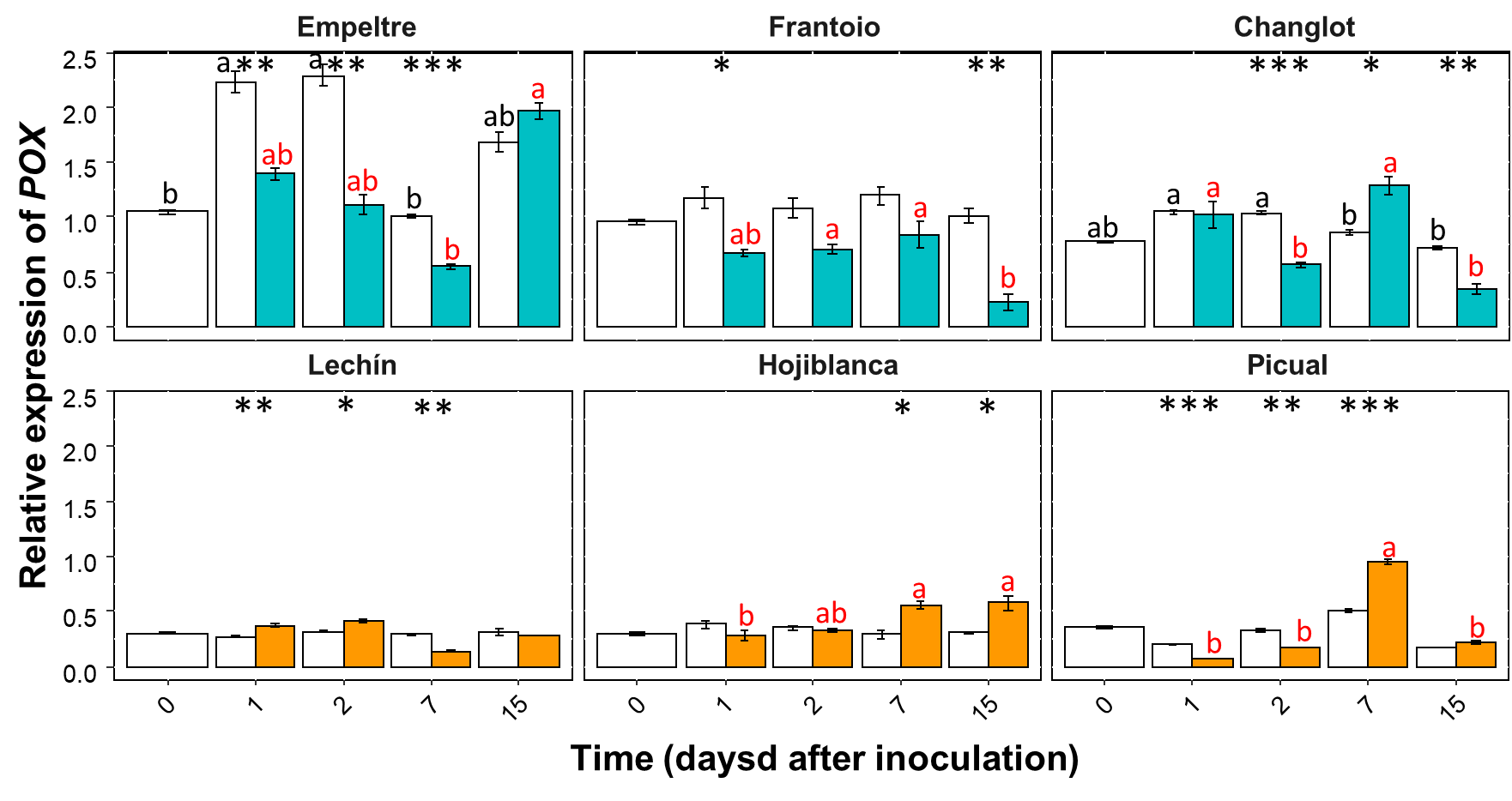


**Supplementary Figure 5** Time course of the relative expression of the *POX* gene. Control (non inoculated) plants are represented in white color while *Verticillium dahlia*e-inoculated plants are shown in light blue color (tolerant cultivars) or in orange color (susceptible varieties). The error bar corresponds to the standard deviation (SD) from the mean of the three biological replicates. Tukey *post hoc* test differences (*p* <0.05) are represented with black letters among control plants and in red letters among inoculated plants. The statistical differences resulted by the ANOVA analysis between control and inoculated plants are represented by asterisks (level of significance: *, *p* < 0.05; **, *p* < 0.01; ***, *p* < 0.001).


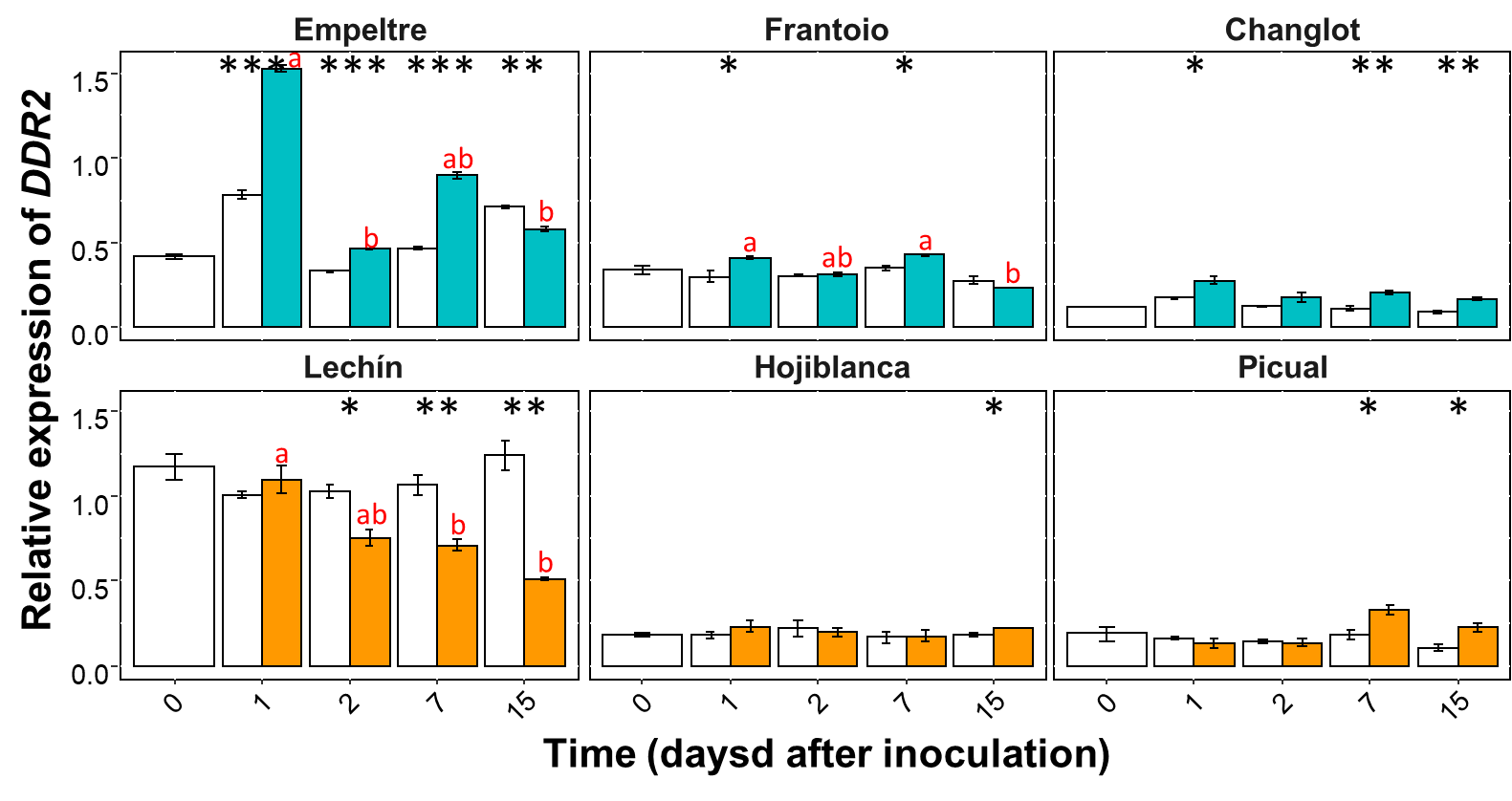


**Supplementary Figure 6** Time course of the relative expression of the *DRR2* gene. Control (non inoculated) plants are represented in white color while *Verticillium dahlia*e-inoculated plants are shown in light blue color (tolerant cultivars) or in orange color (susceptible varieties). The error bar corresponds to the standard deviation (SD) from the mean of the three biological replicates. Tukey *post hoc* test differences (*p* <0.05) are represented with black letters among control plants and in red letters among inoculated plants. The statistical differences resulted by the ANOVA analysis between control and inoculated plants are represented by asterisks (level of significance: *, *p* < 0.05; **, *p* < 0.01; ***, *p* < 0.001).
